# Inhibition of the CCL2 receptor, CCR2, enhances tumor response to immune checkpoint therapy

**DOI:** 10.1038/s42003-020-01441-y

**Published:** 2020-11-27

**Authors:** Megan M. Tu, Hany A. Abdel-Hafiz, Robert T. Jones, Annie Jean, Katelyn J. Hoff, Jason E. Duex, Ana Chauca-Diaz, James C. Costello, Garrett M. Dancik, Beth A. Jirón Tamburini, Bogdan Czerniak, Jonathan Kaye, Dan Theodorescu

**Affiliations:** 1 Department of Surgery, University of Colorado Anschutz Medical Campus, Aurora, CO USA; 2 Department of Medicine, Cedars-Sinai Medical Center, Los Angeles, CA 90048 USA; 3 Cedars-Sinai Samuel Oschin Comprehensive Cancer Institute, Los Angeles, CA USA; 4 Department of Pharmacology, University of Colorado Anschutz Medical Campus, Aurora, CO USA; 5 Department of Medicine, School of Medicine, University of Colorado Anschutz Medical Campus, Aurora, CO USA; 6 Department of Computer Science, Eastern Connecticut State University, Willimantic, CT USA; 7 Department of Pathology, The University of Texas MD Anderson Cancer Centre, Houston, TX USA; 8 Research Division of Immunology, Departments of Biomedical Sciences and Medicine, Cedars-Sinai Medical Center, Los Angeles, CA 90048 USA; 9 Department Surgery, Cedars-Sinai Medical Center, Los Angeles, CA 90048 USA

**Keywords:** Cancer immunotherapy, Chemokines

## Abstract

Immunotherapies targeting the PD-1/PD-L1 axis are now a mainstay in the clinical management of multiple cancer types, however, many tumors still fail to respond. CCL2 is highly expressed in various cancer types and has been shown to be associated with poor prognosis. Inhibition or blockade of the CCL2/CCR2 signaling axis has thus been an area of interest for cancer therapy. Here we show across multiple murine tumor and metastasis models that CCR2 antagonism in combination with anti-PD-1 therapy leads to sensitization and enhanced tumor response over anti-PD-1 monotherapy. We show that enhanced treatment response correlates with enhanced CD8^+^ T cell recruitment and activation and a concomitant decrease in CD4^+^ regulatory T cell. These results provide strong preclinical rationale for further clinical exploration of combining CCR2 antagonism with PD-1/PD-L1-directed immunotherapies across multiple tumor types especially given the availability of small molecule CCR2 inhibitors and antibodies.

## Abstract (web summary)

Investigating signalling induced by the cytokine CCL2 as a therapeutic target, Tu et al demonstrate that blockade of the CCL2 receptor, CCR2 enhances CD8+ T cell recruitment and activation and the therapeutic efficacy of PD-1 inhibition in tumours.

## Introduction

Advances in and approvals for the use of immune checkpoint-based therapies for the treatment of cancer has led a new wave of available second-, and more recently, first-line therapies to traditional chemotherapy and radiation regimens. Programmed cell death protein 1 (PD-1) directed therapies have been shown to re-invigorate CD8^+^ T cells, rescuing them from an exhausted state, leading to a renewed immune response and in many cases, durable clearance of the tumor^1^. Targeting PD-1 both as a monotherapy or in combination with chemotherapy, targeted agents, or other types of immunotherapy^2–9^ has been or is being tested in over 3000 clinical trials^10^. While many of the patients in these trials have improved responses relative to standard of care, a significant number will not benefit from such therapies^10^. This has led to a focus on identifying more effective therapeutic combinations^11,12^.

The development of new anti-cancer drugs targeting cytokines and/or their cognate receptors has been an area of great interest in cancer treatment, whether for monotherapy or as an adjuvant in combination with other therapeutic agents. The use of IL-2 to activate the immune system of cancer patients was an early milestone in current cancer immunotherapy. IL-2-based immunotherapy proved that the immune system could eradicate tumor cells^13–15^. While relatively infrequent, complete remission and long-term disease-free survival has been reported in patients with melanoma and renal cell carcinoma following IL-2 treatment^16^.

To date, the majority of the novel combinations that have or are being tested clinically have involved empirical combinations of immune checkpoint-based therapies, with each other as well as with other agents in complementary therapeutic classes^11,12^. Newer approaches that identify successful combinations are based on an understanding of the mechanisms underlying the failure of patients to respond to monotherapy^17,18^, as well as studies employing functional genomic screens in experimental models^19,20^. Using an in vivo functional genomics screen, we identified the DDR2 receptor tyrosine kinase as an important determinant of the efficacy of immune checkpoint blockade therapy, where inhibition in combination with PD-1-targeted antibodies leads to enhanced tumor response compared to monotherapy^21^. Similarly, Manguo et al.^20^ performed a loss-of-function in vivo genetic screen which identified PTPN2 as a potential target for enhancing immunotherapy. In another screen, Patel et al.^19^ identified *APLNR* as a critical gene for enhanced response to immunotherapy, where the loss of APLNR reduced the efficacy of T cell-based immunotherapies.

One of the more compelling candidates from our screen was the cytokine, CCL2/MCP-1, monocyte chemotactic and activating factor/monocyte chemoattractant protein-1^21^. CCL2 is a key molecule in macrophage chemotaxis and activation^22^, and is implicated in the pathogenesis of several diseases including psoriasis, rheumatoid arthritis, asthma, and atherosclerosis^23–25^. Blockade of CCL2/CCR2 signaling provides protective immunity in murine models of OVA-induced allergic asthma^26^. CCL2, produced by both cancer and stromal cells, preferentially binds to CCR2, which is expressed to varying degrees in a wide range of organs and tissues including blood, brain, heart, kidney, liver, lung, ovary, pancreas, spinal cord, spleen, and thymus. High levels of CCL2 have been identified in patients with lung adenocarcinoma who traditionally have poorer prognosis^27^. Elevated expression of tumor and systemic CCL2 is associated with poor prognosis in breast cancer patients^28,29^. CCL2 is also overexpressed in human liver cancers and is prognostic for hepatocellular carcinoma patients^30^. Interactions between CCL2/CCR2 have been shown to recruit immunosuppressive cells such as myeloid-derived suppressor (MDSC) cells and metastasis-promoting monocytes^31,32^. There is also compelling evidence for the targeting of CCL2/CCR2 in the treatment of various cancers. Knockout or blockade of CCL2/CCR2 inhibits primary liver tumor and metastatic growth leading to prolonged survival^30^.

Here we examined the biological significance of our screen findings^21^ which indicated blockade of the CCL2 receptor, CCR2, enhances the therapeutic efficacy of PD-1 inhibition in tumors. We confirmed, in multiple cancer types, that the combination anti-PD-1 and CCR2-targeted therapy leads to enhanced efficacy compared to either agent alone. Exploring the mechanisms of action underlying this finding revealed distinct differences in the immune cell populations and cytokine profile of the combination-treated tumors compared to that of monotherapies.

## Results

### Identification of CCL2/CCR2 as candidate for enhanced response to anti-PD-1

We recently reported a functional genomics study evaluating selected U.S. Food and Drug Administration (FDA)–approved drugs, whose corresponding target genes, when inhibited could potentiate the response to anti–PD-1 immunotherapy^21^. In this study^21^, a murine bladder tumor cell line was established from N-butyl-N-(4-hydroxybutyl)nitrosamine (BBN)-induced tumors and adapted to in vitro cell culture to be used for screening purposes. The cells were utilized in an in vivo shRNA-based screen identified genes, that when knocked-down, showed enhanced tumor cell death mediated by the immune-activating anti-PD-1^21^. To determine this, tumor samples were sequenced to quantify shRNA constructs which were absent and present, comparing between the isotype control and anti-PD-1-treated groups, with a goal of identifying genes that are preferentially lost in the anti-PD-1-treated^21^. The study discovered and then validated DDR2 kinase as a promising target leading to the enhancement of response to anti-PD-1 immunotherapy^21^.

In addition to DDR2, several of the other genes, when depleted by shRNA seemed to enhance the effect of anti–PD-1 immunotherapy and thus may also be viable targets to consider. Hence, we began a multistep process to select additional targets from this screen for further investigation.

**Fig. 1:**
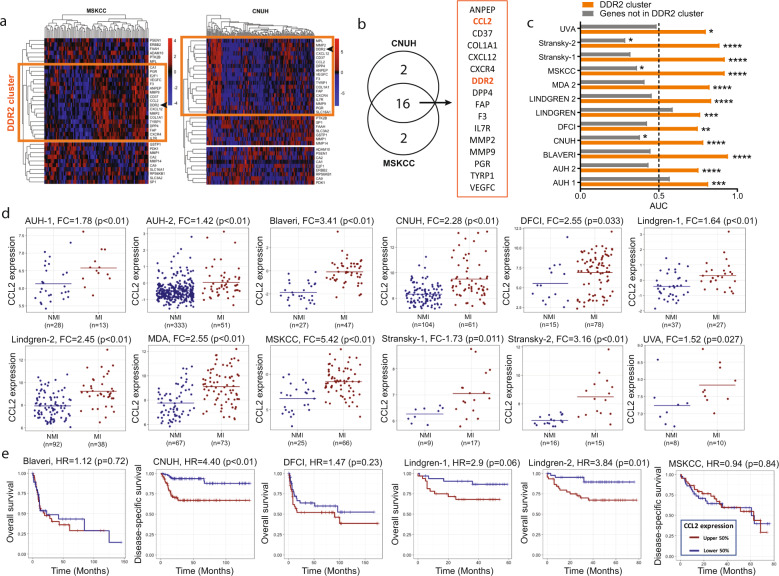
Association of CCL2 expression with stage and outcome in bladder cancer. **a** Unsupervised clustering of the genes from the functional genomics screen^21^ in MSKCC (*n* = 91 independent patient samples) and CNUH (*n* = 165 independent patient samples) cohorts identified three groups of highly correlated genes in each cohort. DDR2 was in one of these groups (orange boxes). **b** The overlap between the groups encompassing DDR2 in each cohort identified a 16 gene DDR2 consensus set (called “DDR2 cluster”). **c** Ability of the 16 gene DDR2 cluster score (average normalized expression of all cluster genes) to distinguish between patients with non-muscle invasive (NMI) and muscle-invasive (MI) tumors in 12 patient cohorts (*n* = 1257 independent patient samples). The length of each bar corresponds to the area under the receiver operating characteristic (ROC) curve (AUC), with AUC > 0.50 indicating a higher DDR2 cluster score in patients with MI tumors. Orange bar represents DDR2 cluster and gray bar represents all genes not in DDR2 cluster. Dotted line denotes AUC = 0.50, or what is expected by random chance. *P* values shown are calculated using the Wilcoxon rank-sum test. **d** Dot plots showing CCL2 expression in NMI and MI bladder tumors in 12 patient cohorts (*n* = 1257 independent patient samples). For each cohort, Fold Change (FC) and *p* values calculated by non-parametric Wilcoxon rank-sum test are reported. **e** Kaplan–Meier curves stratifying patients by high (red curves) and low (blue curves) CCL2 expression, relative to the median cut-point in six patient cohorts (*n* = 505 independent patient samples). Hazard ratios (HR) and log-rank *p* values are reported. Differential expression was evaluated using the non-parametric Wilcoxon rank-sum test to assess statistical significance. Survival analysis was carried out by generating Kaplan–Meier curves, reporting the hazard ratio (HR) and calculating *p*-values using the log-rank test by fitting cox proportional hazard models in *R*.

First, we looked for genes whose expression pattern is consistently associated with the expression pattern of DDR2 in human tumors since these may conceivably be members of signaling pathways that a tumor could use to escape DDR2 depletion. Using two human bladder cancer datasets, CNUH^33^ and MSKCC^34^, we identified 18 genes in each dataset whose expression pattern grouped with that of DDR2 (Fig. 1a). Interestingly, 16 of the 18 genes (80%) grouped with DDR2 in both datasets (*p* < 0.001 by Fisher’s Exact Test) (Fig. 1b) and from here on will be called the “DDR2 cluster”.

To further determine the clinical relevance of this gene set, we developed an expression score from these 16 genes in the DDR2 cluster, and found this to be statistically significantly different in patients with less aggressive non-muscle invasive (NMI) compared to aggressive and advanced muscle-invasive (MI) tumors across 12 different bladder cancer patient cohorts (Fig. 1c). An area under the curve (AUC) < 0.50 indicates higher expression in NMI tumors, while an AUC > 0.50 indicates higher expression in MI tumors, and an AUC = 0.5 is what is expected by chance. High expression score of the 16 gene DDR2 cluster is associated with the more advanced MI disease (Fig. 1c). In contrast, a score generated from the genes outside the DDR2 cluster has an AUC < 0.5 in 10 of the 12 datasets indicating that these genes are not associated with the generally non-fatal NMI disease (Fig. 1c).

Since PD-1 treatment alone does not affect tumor growth^21^, we reasoned that any genes among the 16 that are higher in the PD-1-treated tumors compared to IgG controls, but unchanged or lower in responder tumors harboring shDDR2 and PD-1 treated, could conceivably be ideal additional targets for therapy. This gene selection strategy implies the expression of these genes may contribute to resistance to anti-PD-1 treatment. To determine if such genes exists, we grew control and shRNA depleted NA13 tumors, treated them with IgG or anti-PD-1 (Supplementary Fig. 1) and carried out RNA-seq as described^21^. Interestingly, three genes survived these strict selection criteria: fibroblast activation protein alpha (FAP), CCL2 and CXCR4 (Table 1). Interestingly, while CCR2 is the primary receptor for CCL2^35^, this ligand can also bind and activate CXCR4^36^. Given the implication of macrophages via CCL2 as contributors to metastatic bladder cancer^37^ and potential for immunotherapy^38^, this was of significant interest.

**Table 1:** RNA-seq analysis of in vivo-grown control and shRNA-mediated depletion of DDR2 NA13 tumors, treated with IgG or anti-PD-1.

Gene	log2 Fold Change-Ctrl PD-1 vs Ctrl IgG	*P* value	FDR	log2 Fold Change-shDDR2 PD-1 vs Ctrl PD-1	*P* value	FDR
*Adam10*	−0.822596659	0.020806363	0.045585636	0.406506375	0.48972383	0.69464212
*Anpep*	−1.59767458	1.97872E-05	4.33527E-05	2.158521221	0.000295296	0.000418858
*Ccl2*	1.571613003	2.14919E-07	4.70876E-07	−1.288996781	0.021993815	0.031196828
*Cd37*	−0.042166571	0.941459914	0.9999	1.410860957	0.058872555	0.083506977
*Col1a1*	−0.167174285	0.704017938	0.9999	0.80177797	0.341239809	0.484026975
*Cxcl12*	0.60349953	0.094849721	0.207810699	0.50336306	0.379091454	0.537717127
*Cxcr4*	0.650687462	0.034177217	0.074880467	−0.306546059	0.572535787	0.812105616
*Dpp4*	1.338848907	0.022922195	0.050221311	0.34210444	0.649167245	0.920802468
*E2f1*	−0.010405546	0.967081161	0.9999	0.158559888	0.790202949	0.9999
*Erbb2*	−0.849235451	0.009699223	0.021250482	0.31783298	0.577566925	0.819241966
*F3*	−0.370653036	0.270870723	0.593463358	−0.159093063	0.763568714	0.9999
*Faah*	−0.439111486	0.557325622	0.9999	1.216752365	0.116775867	0.165639143
*Fap*	1.630442692	4.19435E-05	9.1896E-05	−0.893607113	0.122541449	0.173817256
*Gstp1*	0.439742236	0.199664558	0.437454436	−0.460375803	0.369549503	0.524182477
*Il7r*	−0.278499577	0.583310576	0.9999	1.313357477	0.035733225	0.050685308
*Mmp14*	−0.32431483	0.323697891	0.709204875	0.410117128	0.48862662	0.693085797
*Mmp2*	−0.613897569	0.076753939	0.168163802	0.972047317	0.079696089	0.113043836
*Mmp9*	0.083811224	0.812235689	0.9999	−0.21140455	0.697798397	0.989782665
*Pdk1*	0.114935953	0.708139487	0.9999	−0.349615903	0.531679849	0.754154066
*Ptk2b*	−0.796466967	0.04643294	0.101732105	0.402383049	0.487882786	0.692030716
*Rps6kb1*	−0.17291805	0.556540513	0.9999	−0.150150265	0.773998339	0.9999
*Slc3a2*	−0.262119972	0.434579646	0.952140908	0.320402368	0.589868823	0.836691427
*Vegfc*	0.037866013	0.965403498	0.9999	0.037600307	0.964890214	0.9999

‘Ctrl PD-1 vs Ctrl IgG’ depicts comparisons between scramble shRNA control treated with anti-PD-1 vs. IgG control. ‘shDDR2 PD-1 vs. Ctrl PD-1’ depicts DDR2- targeting shRNA vs scramble shRNA control treated with anti-PD-1.

To evaluate this further in human tumors, we used the BC-BET tool^39^, and found that CCL2 expression is higher in patients with MI compared to NMI tumors in all 12 datasets examined (Fig. 1d). High CCL2 expression is also associated with worse disease-specific or overall survival in multiple datasets (Fig. 1e). Interestingly, it has been shown that CCL2 expression is associated with resistance to anti-PD-1 therapy^21^. Taken together, these data suggest a strong rationale for investigating the efficacy of combination CCL2 inhibition with anti-PD-1 therapy in models of cancer.

### Targeting of CCL2 sensitizes primary murine bladder tumor growth to anti-PD-1 therapy

**Fig. 2:**
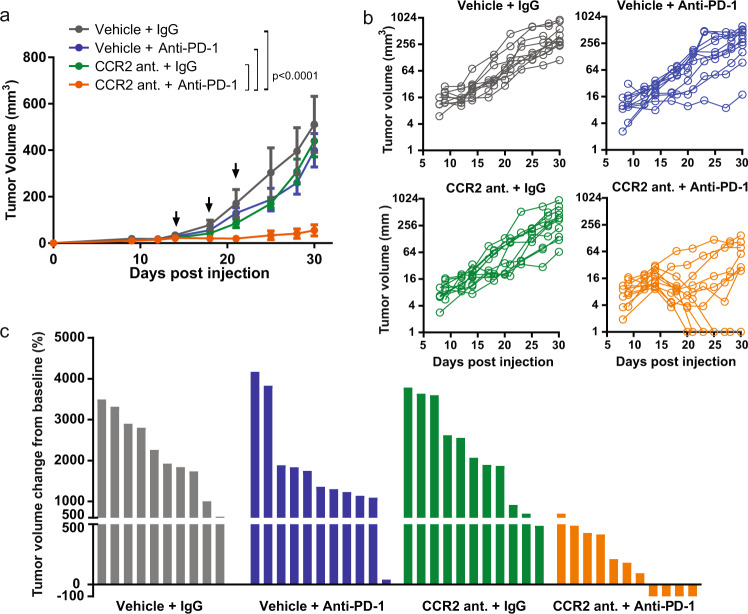
In vivo effect of targeting CCL2 or CCR2 in combination with anti-PD-1 immunotherapy. **a** Subcutaneous tumor growth in syngeneic mice injected with NA13 cells (*n* = 6 biologically independent mice per group). Data representative of two independent experiments. Statistical significance was determined by two-way ANOVA. Arrows indicate dates in which anti-PD-1 treatment was given. Mean ± SD. **b** Individual tumor volumes as a function of time. Each line represents a single mouse (*n* = 10 or 11 biologically independent mice per group). **c** Waterfall plot showing change in NA13 tumor volume on endpoint day 30 post tumor injection compared to baseline prior to treatment with and without CCR2 antagonist RS504393 and/or anti-PD-1 treatment. Starting on day 14, mice were treated daily with CCR2 antagonist RS503393, and every three days with anti-PD-1.

We looked to test the efficacy of targeting CCR2 in combination with PD-1 in multiple tumor models. The tumor cell lines, NA13, B16F10 and E0771, express CCL2 and PD-L1 in vitro (Supplementary Fig. 2) and PD-L1 in vivo (Supplementary Fig. 3) to varying levels. To determine the impact of reduced CCL2 activity on tumor growth when combined with anti-PD-1, we depleted CCL2 expression in NA13 cells using either of two different shRNAs (Supplementary Fig. 4a). Knockdown of CCL2 did not affect growth of NA13 in vitro (data not shown). NA13 cells expressing CCL2 shRNA (shCCL2) were inoculated into mice and on day 14 received treatment with IgG control or anti-PD-1. Tumor growth is hindered in the shCCL2 tumors that received anti-PD-1, but not in the shCCL2 tumors receiving IgG (Supplementary Fig. 4b). To determine the translational potential of this approach we targeted CCR2 using RS504393, a small molecule inhibitor of the CCL2 cognate receptor^40,41^. RS504393 is a highly selective CCR2 chemokine receptor antagonist used frequently in the prevention of CCL2/CCR2 interactions in vitro and in in vivo murine models^42–48^. When RS504393 is utilized in combination with anti-PD-1 immune checkpoint blockade, this limited NA13 tumor growth synergistically, and in some cases, this resulted in complete tumor clearance (Fig. 2).

### Combination therapy with CCR2 antagonist RS504393 and anti-PD-1 improves therapeutic efficacy of murine melanoma pulmonary metastases

**Fig. 3:**
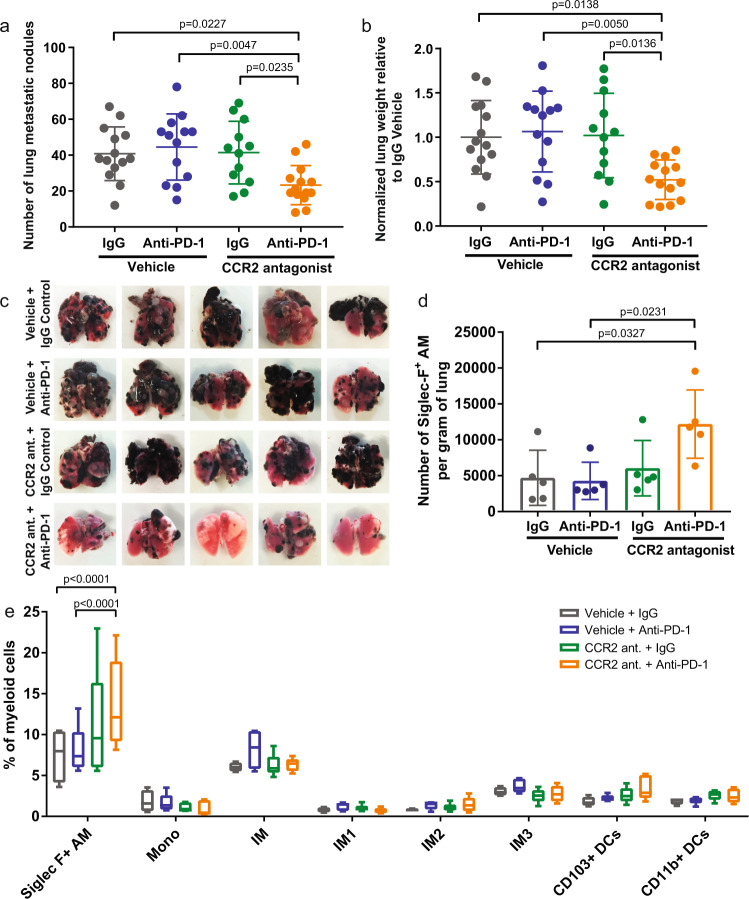
Combination treatment with CCR2 antagonist RS504393 and anti-PD-1 controls lung metastatic nodules. **a** Visual quantification by ×10 magnification of metastatic B16F10 lung nodules at euthanasia on day 14 post tumor cell injection. Each dot represents a biologically independent mouse. Mean ± SD. Statistical significance determined by one-way ANOVA. **b** Lung weight of mice bearing B16F10 lung metastases. Each dot represents a biologically independent mouse. Mean ± SD. Statistical significance determined by one-way ANOVA. **c** Representative images of murine pulmonary lung metastases at 28 days following intravenous (tail vein) inoculation of B16F10. Flow cytometric analysis of lung infiltrating (**d**) alveolar macrophages and (**e**) immune cell subsets. Box plot with min/max whiskers. *n* = 5 biologically independent mice per group. Statistical significance determined by two-way ANOVA. If statistical significance not indicated in the figure, *p* > 0.05. Alveolar macrophage (AM), monocyte (Mono), interstitial macrophage (IM), interstitial macrophage subtype 1 (IM1), interstitial macrophage subtype 2 (IM2), interstitial macrophage subtype 3 (IM3), and dendritic cell (DC).

To assess the efficacy of CCR2 antagonist and anti-PD-1 combination treatment in models beyond bladder cancer as well as in metastatic disease, we examined the efficacy of this combination in the treatment of B16F10 melanoma pulmonary metastases. B16F10 is known to be poorly controlled by PD-1-based monotherapy^49–51^. In support of our hypothesis, we found that combination therapy is effective in reducing the number of pulmonary metastases compared to the control or mono therapy cohorts (Fig. 3a–c). These differences were confirmed through lung mass quantification wherein the control and single treatment groups exhibited higher tumor mass burden (Fig. 3b).

To better understand the underlying mechanism accounting for the differences in tumor burden, we looked at the lung tumor microenvironment using flow cytometry (Supplementary Fig. 5). Analysis of the lungs from the four treatment cohorts indicate a difference in the Siglec-F^+^ alveolar macrophage (AM) population, while no statistically significant differences were seen in the monocytes (Mono), interstitial macrophages (IM) or dendritic cell (DC) populations (Fig. 3d, e). Siglec-F, used in conjunction with CD11b and CD11c, is a marker specific for murine lung-resident AMs that is not expressed by interstitial or inflammatory macrophages^52,53^. The statistically significant higher ratio of AMs in the combination-treated group (Fig. 3d, e), which had the lowest tumor burden, is in agreement with previous observations in which higher AM presence is associated with lower lung tumor burden^54^. Siglec-F^+^ AMs preferentially remain localized in the healthy alveolar space outside of the tumor nodules and are progressively reduced as tumor burden increases^54^. Thus, their greater presence in the combination-treated group provides further support to our observations of lower tumor burden in this group.

### CCR2 antagonism also enhances response to anti-PD-1 therapy in orthotopically implanted murine mammary tumors

**Fig. 4:**
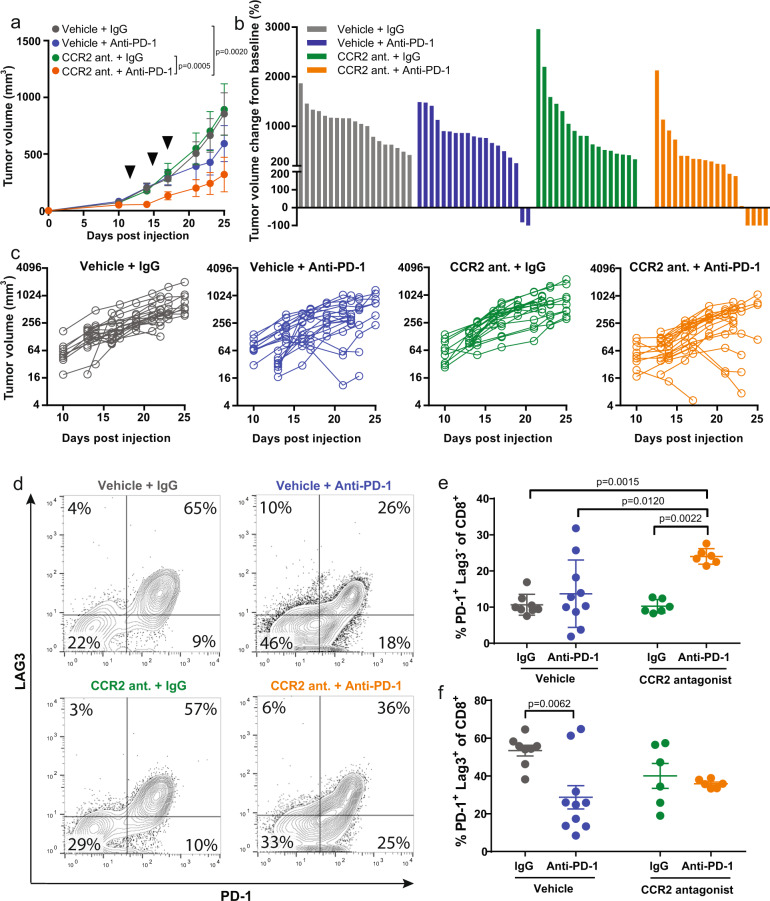
Combination CCR2 antagonist RS504393 and anti-PD-1 suppresses mammary tumors. **a** Mammary fat pad tumor growth in syngeneic mice injected with E0771 (*n* = 8 or 9 biologically independent mice per group). Data representative of three independent experiments. Arrows indicate dates in which anti-PD-1 treatment was given. **b** Waterfall plot showing change in E0771 tumor volume compared to baseline prior to treatment. **c** Individual tumor volume as a function of time. Each line represents a single mouse (*n* = 17 or 19 biologically independent mice per group). **d** Representative image of raw flow cytometry data looking at LAG3 and PD-1 expression on tumor-infiltrating CD8^+^ T cells. Percentage of (**e**) PD-1^+^ LAG3^-^ and (**f**) PD-1^+^ LAG3^+^ of CD8^+^ T cells. Mean ± SD. Statistical significance was determined by two-way ANOVA. If statistical significance is not indicated in the figure, *p* > 0.05.

**Fig. 5:**
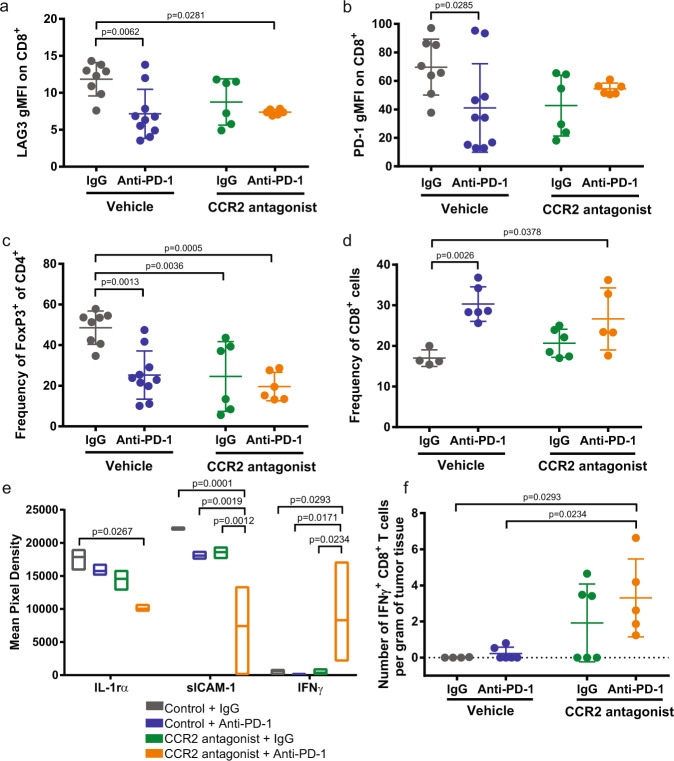
Immune population and cytokine analysis of tumors. Flow cytometry-based analysis of (**a**) LAG3 and (**b**) PD-1 expression levels on CD8^+^ T cells from E0771 tumors. Frequency of (**c**) FoxP3^+^ CD4^+^ and (**d**) CD8^+^ T cells of tumor-infiltrating lymphocytes. **e** Cytokine array of tumor tissue harvested 25 days post subcutaneous E0771 injection into mice. Data are presented as mean pixel density as determined on the membrane by chemiluminescence with Biorad ChemiDoc MP Imaging system. **f** Number of IFNγ^+^ CD8^+^ T cells per gram of tumor tissue. All dot plots are presented as mean ± SD and box plots as mean with min/max. Statistical significance was determined by two-way ANOVA.

Since the pro-tumorigenic role of the CCR2/CCL2 axis has been widely reported in breast cancer^28,29,32,47,55^, we looked to test the combination therapy in a breast cancer model utilizing the E0771 cell line, which was originally derived from a spontaneous mammary tumor in a female C57BL/6 mouse^56^. Once again, combination therapy with CCR2 antagonist and anti-PD-1 is more efficacious than monotherapy in reducing tumor growth (Fig. 4a–c). Flow cytometry-based analysis of the tumors reveal differences in the expression level of the T cell exhaustion markers LAG3 and PD-1 (Fig. 4d–f, Supplementary Fig. 6). A higher proportion of PD-1^+^ LAG3^−^ on CD8^+^ T cells were identified in the tumors from mice treated with CCR2 antagonist and anti-PD-1 (Fig. 4e). In contrast, no statistically significant differences were seen in the PD-1^+^ LAG3^+^, PD-1^−^ LAG3^−^, and PD-1^−^ LAG3^+^ populations between untreated control and combination therapy (Fig. 4f, Supplementary Fig. 7). Overall expression levels of LAG3 and PD-1 are reduced following treatment with CCR2 antagonist and/or anti-PD-1 compared to control (Fig. 5a, b). The PD-1-treated and combination-treated tumors have lower levels of PD-1^+^ LAG3^+^ TIGIT^+^ CD8^+^ T cells (Supplementary Fig. 8a), though no differences were seen based on the expression of TIGIT alone (Supplementary Fig. 8b). Furthermore, the frequency of inhibitory FoxP3^+^ CD4^+^ regulatory T cells are reduced following single agent and combination treatment compared to control, though more statistically significant in the combination-treated group (Fig. 5c). The frequency of CD8^+^ T cells varies amongst the tumors from the four different treatment cohorts, with the control group possessing the lowest frequency of CD8^+^ T cells (Fig. 5d). The combination-treated group possesses a higher ratio of PD-1^+^LAG3^−^ and IFNγ^+^ CD8^+^ T cells relative to Tregs (Supplementary Fig. 9). All three of the tumor cell lines, NA13, B16F10, E0771, express class I MHC at various levels indicating that they can be direct targets of CD8^+^ T cell recognition (Supplementary Fig. 10a). E0771-OVA cells expressing the ovalbumin peptide SIINFEKL were utilized to determine whether the CD8^+^ T cell response is tumor-specific. Tumor-infiltrating cells were stained with a tetramer to identify CD8^+^ T cells that recognize the tumor antigen. There is an increased average number of tetramer^+^ CD8^+^ T cells in the tumors from the combination-treated group, though not statistically significant. Of note, three of the five mice show a very strong response with increased recruitment of antigen-specific T cells with combination therapy (Supplementary Fig. 10b). No differences are seen in the frequency of monocytes and macrophages between control and combination-treated tumors. (Supplementary Fig. 11).

Looking at bulk tumors through a cytokine panel analysis, statistically significant differences were seen in three cytokines across the treatment groups (Fig. 5e, Supplementary Fig. 12). Tumors from mice treated with combination therapy exhibit lower levels of IL-1rα and sICAM-1, but higher levels of IFNγ (Fig. 5e, Supplementary Fig. 12). Furthermore, while no differences are seen in the frequency of IFNγ^+^ CD8^+^ T cells, the raw number of cells per gram of tumor reveals a higher number of IFNγ^+^ CD8^+^ T cells in the combination-treated group when comparing on an equal tumor weight basis (Fig. 5f). Next, we performed CIBERSORT analysis to estimate relative immune cell populations of human bladder cancer RNA sequencing data from TCGA^57^ (Supplementary Fig. 13). In comparing immune population differences between the bottom and top quartile of CCL2 tumor expression, a higher presence of activated DCs and natural killer cells, both producers of IFNγ, are observed in the CCL2 low tumors (Supplementary Fig. 13). While CD8^+^ T cells are inferred to be higher in the CCL2 high tumors, analysis with CIBERSORT does not account for the activation or exhaustion status of these T cells (Supplementary Fig. 13). Though a higher presence of Tregs are observed in the CCL2 low tumors, inferences between the experimental data and CIBERSORT analysis possess a limitation in the conclusions that can be drawn. A key difference that should be noted is that CIBERSORT is looking at intrinsic, baseline CCL2 levels versus the mouse experiments which are treatment blockade-based. These human data will have considerably more variability/heterogeneity due to the fact that these are inferred estimates of immune populations that come from clinical sequencing samples from a tumor type, MIBC, that is known to have a high amount of inter- and intra-tumoral heterogeneity.

Overall, we observed that tumors from mice treated with combination therapy possessed significant increases in cytotoxic T cell recruitment and activation, with a concomitant decrease in suppressive regulatory T cells. We believe this change in the T cell population to be correlative with the observed reduction in tumor and metastatic burden in combination-treated mice.

## Discussion

Inhibition of CCL2/CCR2 has been explored in the clinical setting as a possible cancer therapeutic. While the available data are still sparse so far, no clear benefit for this therapy has emerged. A phase 1b trial in non-metastatic pancreatic cancer patients suggests that CCR2 inhibition decreases tumor-infiltrating macrophages and regulatory T cells, while also increasing effector T cells^58^. Combination therapy of CCX872, a CCR2 specific antagonist, with FOLFIRINOX (fluorouracil [5-FU], leucovorin, irinotecan, oxaliplatin) suggests better overall survival with the combination compared to monotherapy (29% vs 19% at 18 months weeks) with no safety concerns^59^. In contrast, a phase 1 trial (NCT00537368) of a human IgG1_k_ monoclonal antibody which binds CCL2, carlumab, also known as CNTO888, in patients with advanced solid tumors refractory to conventional treatments showed no objective anti-tumor response in any of the 44 patients enrolled^60^. A phase 2 study (NTC00992186) to assess the efficacy of carlumab in patients with metastatic prostate cancer reported that no patients responded^61^. These human studies are in line with results that we report here, where we observed modest or no responses to monotherapy with CCL2/CCR2 blockade. An important finding common to all these studies is the general tolerability of the treatment with few mild-to-moderate adverse events. This makes CCL2/CCR2 inhibition ideal for combining with other therapies, such as with immune checkpoint blockade.

The improved efficacy of the combination treatment when CCR2 antagonist and anti-PD-1 are used concurrently to target the tumor (Fig. 2) compared to CCL2 knockdown (Supplementary Fig. 4b) could be attributed to several reasons: better reduction in CCR2 receptor signaling that is achieved by reduction in CCL2; blockade of CCL2 being expressed by other cell types, such as CAFs, which are known to secrete CCL2 and promote macrophages in the tumor; and finally, non-specific effects of the inhibitor that serendipitously affect anti-PD-1 response. Since tumor intrinsic CCL2 may not be the only relevant source of CCL2, in our study we chose to target the CCL2 receptor, CCR2. Furthermore, CCR2 antagonists provide greater real-world relevance in which the findings from our study could more readily be applied due to the use of CCR2 antagonists in clinical trials. BMS-741672, BMS-813160, PF-04634817 are three CCR2 antagonists which have been or are currently being tested in clinical trials (ClinicalTrials.gov Identifier: NCT04123379, NCT00699790, NCT01712061, NCT00699790). Indeed, based on our findings, therapy targeting CCL2/CCR2 in combination with blockade of PD-1 may produce synergistic responses in some patients. In addition to blocking CCL2/CCR2 and PD-1 separately, development of bispecific antibodies^62^ would be a novel and elegant solution towards inhibiting these molecules in the cancer cell.

In our analysis of the tumor-infiltrating immune cells, higher proportions of PD-1^+^ LAG3^+^ CD8^+^ T cells were present in the tumors of control mice. Like the immune checkpoint PD-1, LAG3 is a co-inhibitory receptor which suppresses T cell activation and cytokine secretion^63^. LAG3 and PD-1 are commonly described as markers of exhausted T cells with combination targeting of the two being effective in eliciting a strong T cell response leading to tumor clearance^64^. LAG3 has been reported to synergize with PD-1, potentially amplifying the PD-1 inhibitory effect^65^. Thus, it is possible that downregulation of LAG3 by this novel combination treatment might alter responsiveness to immune checkpoint blockade. Recently, progenitor, transitional, and terminally exhausted subsets of CD8^+^ T cells were identified in response to chronic stimulation, with differential responsiveness to checkpoint blockade^66^. In that study, LAG3 was most highly expressed in the terminally exhausted subset. The appearance of PD-1^+^ LAG3^−^ cells correlates with treatment effectiveness, but whether these cells represent a specific type of effector cell, are direct progenitors of effector cells, or are another transitional population of cells will require more extensive analysis of their transcriptome in the future.

While we observed few changes in the myeloid compartment after blocking the CCL2/CCR2 axis combined with or without anti-PD-1, an increase in the Siglec-F^+^ AM population was seen (Fig. 3d, e). Siglec-F is a marker specific for murine lung-resident AMs that is not expressed by interstitial or inflammatory macrophages^67,68^. AMs play an important role in maintaining lung homeostasis^69^. AMs remove clear apoptotic cells, environmental particles, and pathogens by interacting with cells in the alveolar epithelium via cell-surface receptors and cytokines^70^.

Siglecs belong to the transmembrane lectins family that are expressed on immune cells and binds to sialic acids^71^. Cancer cells express high levels of sialic acids that can interact with Siglecs on immune cells^71^. Siglecs can contribute to both facilitation as well as attenuation of anti-tumor activity^72^. Their anti-tumor effect is due to the inhibition of tumor-promoting inflammation^72^. For example, macrophages that express Siglec-1 (CD169) have been shown to present tumor antigens to cytotoxic T cells, and Siglec-1 deficient macrophages result in inhibition of anti-tumor immunity^73^. The function of Siglec-E, the murine equivalent of human Siglec-9, have been tested in subcutaneous syngeneic mouse model. Once tumors are established, Siglec-E deficiency results in more aggressive tumor growth compared to wildtype^74^.

In a study by Poczobutt et al.^75^, multiple subsets of myeloid cells were identified: MacA cells, which bear markers of AMs (Siglec-F^+^/CD11c^+^), MacB1 cells (CD11b^+^/CD64^lo^/CD11c^+^), MacB2 cells (CD11b^+^/CD64^int^/CD11c2), and MacB3 cells (CD11b^+^/CD64^hi^/CD11c^+^). Using data from PRECOG, they reported that MacA represents a resident AM population that is enriched in genes predicting good clinical outcomes^75^.

We observed a higher number of IFNγ^+^ CD8^+^ T cells in the combination-treated group. Concurrently, we also observed increased levels of IFNγ, as measured through a multi-cytokine panel of bulk tumor. In the clinical setting, high IFNγ expression or associated signatures is associated with improved outcomes to immune checkpoint blockade^76,77^. Metastatic non-small cell lung cancer (NSCLC) and urothelial cancer patients who received PD-L1 inhibitor and subsequently exhibited an increased IFNγ gene signature (*IFNγ, CD274, LAG3*, and *CXCL9*) had better overall response rates and longer median progression‐free survival, independent of PD-L1 expression^76^. Moreover, in melanoma and NSCLC patients treated with anti-PD-1 therapies, higher IFNγ protein expression is associated with longer progression-free survival^77^. Importantly, this suggests that the higher observed proportion of IFNγ and IFNγ-expressing cells in combination-treated tumors from our study are indicative of not only reduced tumor growth in mice but is more likely to be clinically translatable to patient outcomes. Taken together, this finding, the known tolerability in patients of CCR2-targeted therapies, and evidence that the combination is more effective across multiple tumor models, we believe clinical trials to be strongly justified.

## Methods

### Cell lines

NA13 cell line was isolated and cultured from N-butyl-N-(4-hydroxybutyl) nitrosamine (BBN) carcinogen-induced bladder tumor of C57BL/6 female mice^21^. E0771 was a gift from Dr. Traci Lyons (University of Colorado). E0771 was maintained in RPMI with 5% fetal bovine serum (FBS). B16F10 was obtained from the American Type Culture Collection (ATCC) through the University of Colorado Tissue Culture Core and maintained in DMEM media with 10% FBS. All lines have been authenticated and tested to be mycoplasma-free. All lines were grown at 37 °C in a humidified atmosphere (5% CO_2_).

### qPCR

Cells were homogenized using QIAshredder (Qiagen), followed by RNA extraction using a RNeasy Plus Mini Kit with gDNA Eliminator (Qiagen). cDNA was synthesized using iScript Reverse Transcription Supermix (Bio-Rad). qPCR was then performed (Quant Studio 6 Flex Real-Time PCR system, Applied Biosystems, USA), using iQ SYBR Green Supermix (Bio-Rad)^78^. The following primer pairs were used: Mouse CCL2: forward 5′ AGTAGGCTGGAGAGCTACAA 3′; reverse 5′ GTATGTCTGGACCCATTCCTTC 3′ and mouse PD-L1: forward 5′ TCCATCCTGTTGTTCCTCATT 3′ Reverse 5′ TCCACATCTAGC ATTCTCACTTG 3′. To determine the changes in mRNA expression as measured by qRT-PCR, the ΔΔ*C*_t_ method was used. Expression was normalized to internal control β-actin.

### Immunoblot analysis

Western Blot analysis was performed using total protein extracted from NA13, B16F10, and E0771 cell lines using RIPA buffer (Sigma, USA) containing protease and phosphatase inhibitors (Roche). Following total protein quantification, equal amounts of protein were separated by 4–20% Mini-PROTEAN^®^ TGX^™^ Protein Gel (BioRad) and transferred to PVDF membrane. The membrane was probed with antibodies against PD-L1 (MAB90781, R&D Systems, USA) or anti-β-actin (13E5, Cell Signaling, USA) Rabbit monoclonal antibodies diluted at 1:1000 in 5% non-fat milk blocking buffer. The blots were imaged using iBright imaging system according to the manufacturer’s instruction (ThermoFisher Scientific, USA).

### Immunohistochemistry analysis

Immunohistochemistry was performed on 5-µm thick sections of formalin-fixed paraffin-embedded (FFPE) NA13 tumors blocks using a Rabbit anti-mouse antibody (D5V3B, Cell Signaling Technology, USA) at a dilution of 1:100. Antigen retrieval was performed according to standardized protocols by heating with 10 mM citrate buffer. The activity of endogenous peroxidase (peroxidase blocking reagent, Dako) were neutralized and non-specific binding were then blocked with goat serum 1:100 in PBS. Tissue sections were incubated with primary antibodies. The slides were then incubated with anti-rabbit HQ secondary antibody followed by anti-HQ HRP linking antibody (Ventana Medical System, USA). Finally, slides were incubated with Discovery ChromoMap DAB reagent. Slides were counterstained with hematoxylin, dehydrated by ethanol and mounted. Slides were imaged on an Olympus BX43 light microscope with ×20 magnification using an Olympus DP26 digital camera.

### Tumor cells pre-treatment with recombinant interferon γ (rIFNγ)

The response of mouse tumor cells to rIFNγ (R&D system) was tested after incubation of 200,000 cells/well in 6-well tissue culture plate in the presence of rIFNγ (50 and 250 unites) for 48 h followed by FACS analysis of MHC class I antigen expression using APC anti-mouse H-2Db and APC mouse IgG2a K isotype control (FC) antibodies (Biolegend).

### In vivo studies

Female C57BL/6 mice (Charles River) were received at 6-week old and allowed to acclimate for at least one week in sterile micro isolator cages with constant temperature and humidity. Mice had free access to food and water. Mice were housed in specific-pathogen-free conditions and cared for in accordance with US National Institutes of Health guidelines, and all procedures were approved by the University of Colorado Denver Animal Care and Use Committee and carried out according to approved protocols.

For NA13, mice were injected with 1 × 10^6^ cells in 100 µL sterile PBS subcutaneously in the hind flank. For the E0771 mammary tumor model, mice were injected with 5 × 10^4^ cells in the third thoracic mammary fat pad. For the B16F10-induced pulmonary metastases model, mice were inoculated intravenously with 2 × 10^5^ B16F10 cells in 100 µL sterile PBS. Mice were examined twice weekly. Tumor measurements commenced from when the tumor was first palpable. Tumor size was determined using an electronic caliper to measure the length and width and calculated by (*L* × *W*2)/2, where *L* is the largest diameter measurement of the tumor and *W* is the shorter perpendicular tumor measurement. Measurements were taken from distinct samples of each tumor-bearing mouse. Animals were randomized into treatment groups ensuring similar average tumor volumes amongst the groups, weighed and identified via ear punch.

Mouse anti-PD-1 antibody (IgG1-D265A) and isotype control (IgG1, clone 4F7) were produced by Bristol-Myers Squibb laboratories (Redwood City, CA) and was formulated in PBS and administered intraperitoneally at a dose of 50 µg/mouse (NA13, E0771) or 100 µg/mouse (B16F10) for a total of three doses. CCR2 small molecule inhibitor RS504393 (Tocris) was given daily at 2 mg/kg by oral gavage to B16F10 mice and intraperitoneally to E0771 and NA13 mice.

### Flow cytometry

B16F10 mice were euthanized 4 weeks after tumor cell injection. Lungs were extracted and the number of visible metastases was quantified, then lungs were processed and analyzed^79^. E0771 tumors were mechanically dissociated in Click’s media in the absence of mercaptoethanol or L-glutamine (Irvine Scientific). Cells were digested for 1 h at 37 °C with 500 units/ml collagenase type II and IV and 20 μg/ml DNase (Worthington Biochemical). The digested tissue suspension was then filtered through a 100 μm strainer. Filtered cells were carefully layered into a centrifuge tube containing 5 ml Lympholyte-M (Cedarlane). The cells were centrifuged at 1500 × *g* for 20 min., then the interface lymphocyte layer was carefully removed. The cells were washed prior to staining. For the CD8 T cell panel, cells were stained with CD8 APC/Cy7 (clone 53-6.7) (1:400), CD3 FITC (clone 17A2) (1:300), CD45 BV510 (clone 30-F11) (1:300), CD44 BV421 (clone IM7) (1:400), PD-1 PE (clone 29F.1A12) (1:200), LAG3 PerCP-Cy5.5 (clone C9B7W) (1:100), and Tigit APC (clone1G9) (1:100). CD8 T cells were gated on live, CD3^+^/CD8^+^ double-positive cells. The cells were then further classified based on the expression of PD-1 and Lag-3. For the macrophage panel, cells were stained with CD45 BV510, F4/80 APC/Cy7 (clone BM8) (1:100), CD11b BV421 (clone M1/70) (1:600), CD64 PerCP-Cy5.5 (clone X54-5/7.1) (1:200), MERTK FITC (clone 2B10C42) (1:100), PD-L1 PE (clone 10F.9G2) (1:200), and Ly-6G APC/Cy7 (clone 1A8) (1:100). Live cells were gated for CD45^+^ cells. Neutrophils were gated by the expression of Ly-6G^hi^/CD11b^+^, and not included in further analysis. Macrophages were gated as F4/80^+^/CD11b^hi^ population and MERTK^hi/^CD64^hi^ population. Monocytes were confirmed with two population gates as F4/80^lo^/CD11b^+^ and MERTK^lo^/CD64^lo^^79–82^. For the tetramer panel, cells were stained with CD8 APC/Cy7, CD35 BV410, CD44 BV421, LAG3 PerCP Cy5.5, PD-1 APC (clone 29F.1A12) (1:100), CD4 FITC (clone GK1.5) (1:200), and MHC-I-SIINFEKL tetramer PE (NIH) (1:200). Live CD8^+^ cells were gated CD44^hi^/tetramer^+^. Cells were further analyzed for expression of PD-1 and Lag-3. IMs are identified based MERTK^hi^ CD64^hi^ CD11b^hi^, and subdivided into three pulmonary IM subtypes: IM1 (CD11c^lo^ CD206^+^ MHCII^lo^), IM2 (CD11c^lo^ CD206^+^ MHCII^hi^), and IM3 (CD11c^hi^ CD206^lo^ MHCII^hi^). All flow cytometry antibodies used were purchased from Biolegend unless otherwise indicated. All samples were run on the CyAn ADP flow cytometer, acquired using Summit software (BD Biosciences), and analyzed using FlowJo software (Tree Star). Flow cytometry data were taken from distinct samples and not the same samples repeatedly measured.

### Intracellular cytokine staining

Cells were isolated from the tissue and treated with or without (controls) phorbol 12-myristate 13-acetate (PMA) (20 ng/ml) (Sigma, St. Louis, MO) plus ionomycin (1 µg/ml) (Sigma, St. Louis, MO) for 4–6 h at 37 °C in the presence of 2 µg/ml of brefeldin A (Adipogen, San Diego, CO) in RPMI + 2.5% FBS. Cells were then stained with CD8 APC/Cy7, CD45 BV510, CD44 BV421, and CD4 PerCP-Cy5.5 (clone GK1.5) (1:400). Cells were incubated at 37 °C for 30 min. Following incubation, cells were washed, then fixed with 1% paraformaldehyde and 4% sucrose for 10 min at room temperature in the dark. Cells were then permeabilized with BD Perm Wash (BD Biosciences) and stained for cytokines IFNγ APC/Cy7 (XMG1.2) (1:200). Cells were incubated overnight at 4 °C in the dark, then washed with Perm Wash and resuspended in FACs buffer (0.5% Bovine Serum Albumin and 0.1% Sodium Azide in PBS).

For FoxP3 analysis, cells were stained for CD8 APC/Cy7, CD45 BV510, CD4 perCP-Cy5.5, B220 FITC (clone RA3-6B2) (1:300), CD25 APC (clone 3C7) (1:200), and PD-1 BV421 (clone 29 F.1A12) (1:100). Cells were incubated in antibody at 37 °C for 30 min. Cells were fixed and permeabilized for 30 min at 4 °C in the dark using the FoxP3 Transcription Factor Staining Buffer Set (eBioscience; 00-5523). The cells were then stained with FoxP3 PE (MF-14) (1:200) and incubated in the dark overnight at 4 °C. Cells were gated for live CD45^+^, followed by gating for the CD4^+^/CD8^−^ population. Further classification of activated CD4 T cells as CD25^+^/FoxP3^−^ and regulatory CD4 T cells as FoxP3^+^.

### Tumor expression analysis (RNAseq)

Library preparation and sequencing were performed by Novogene^21^. Sequencing was performed on an Illumina platform paired end 150 bp with 20 million reads per sample.

Transcript quantification was done using RSEM (v1.2.31)^83^ with default parameters and Bowtie2 (v2.1.0) as the read aligner^84^. Reads were mapped directly to mouse transcripts and summarized at the gene level using annotations from Ensembl r91, genome build GRCm38.p5. Quantification of genes as expected counts were compiled. Differential expression was performed using voom function in the limma R package^85^. Genes with an average expected count <5 were removed, normalization factors were calculated, and comparisons between groups were made using the voom function using default parameters.

### Protein cytokine array

Tumors were isolated and flash frozen in liquid nitrogen. Tissue was homogenized in PBS containing complete mini protease inhibitor cocktail (MilliporeSigma). Following homogenization, Triton X-100 was added to a final concentration of 1%, then frozen at –80 °C overnight. Samples were thawed on ice and centrifuged at 10,000 × *g* for 5 min, and supernatants were collected. A BCA protein assay was performed to determine lysate protein concentration (Pierce, Thermo Scientific). Purified protein lysates were applied to the Proteome Profiler Mouse Cytokine Array Kit, Panel A as per manufacturer’s instructions (R&D Systems, catalog ARY006). The chemiluminescence reaction was measured with Biorad ChemiDoc MP Imaging system.

### Gene expression analysis

Preliminary analysis using the Bladder Cancer Biomarker Evaluation Tool (*BC-BET*)^39^ was used to evaluate the association of CCL2 expression with bladder cancer characteristics. The 12 patient cohorts (*n* = 1257) included in *BC-BET* were then downloaded^39^. In brief, processed data were downloaded from the Gene Expression Omnibus^86^ (AUH-1 cohort: Accession #GSE3167^87^; AUH-2: GSE5479^88^; CNUH, GSE13507^33^; DFCI: GSE31684^89^; Lindgren: GSE19915^90^; Lindgren-2: GSE32548^91^; MDA-1: GSE48276 and MDA-2: GSE48075^92^; UVA: GSE37317^93^, from Array Express^94^, (Stransky-1 and Stransky-2: E-TABM-147^95^ or as Supplemental Material to Blaveri^96^ and MSKCC^34^. Probes for target genes were identified from the microarray platform annotation. When multiple probes for a gene were available, probe with the highest mean expression was used^97^. Hierarchical agglomerative clustering with complete linkage was used, based on the Euclidian distance between samples. The DDR2 cluster score was calculated by normalizing the expression of each of the 16 probes to have a mean of 0 and a standard deviation of 1, and then finding the mean of all signature probes for each sample.

### CIBERSORT

CIBERSORT^98^ using TCGA bladder cancer gene expression^57^ was processed according to The Cancer Immune Atlas (TCIA) and the associated data as downloaded directly from the TCIA website^99^.

### Statistics and reproducibility

The differences between the groups were evaluated by Student’s *t* test, one-way or two-way ANOVA using GraphPad Prism 7.0 Software. The specific type of statistical analysis utilized is indicated in the corresponding figure legends. Exact *p*-values are indicated in the figure. A value of *p* < 0.05 was considered to be statistically significant. Experiments were repeated at least two times to ensure reproducibility.

### Reporting summary

Further information on research design is available in the Nature Research Reporting Summary linked to this article.

## Supplementary information

Supplementary Information

Description of Additional Supplementary File

Supplementary Data 1

Reporting Summary

## Data Availability

Raw data for graphs can be found in Supplementary Data 1. All other data are available within the manuscript files or from the corresponding author upon reasonable request.

## References

[1] Wei SC, Duffy CR, Allison JP (2018). Fundamental mechanisms of immune checkpoint blockade therapy. Cancer Discov..

[2] Rizvi NA (2016). Nivolumab in combination with platinum-based doublet chemotherapy for first-line treatment of advanced non-small-cell lung cancer. J. Clin. Oncol..

[3] Langer CJ (2016). Carboplatin and pemetrexed with or without pembrolizumab for advanced, non-squamous non-small-cell lung cancer: a randomised, phase 2 cohort of the open-label KEYNOTE-021 study. Lancet Oncol..

[4] Gandhi L (2018). Pembrolizumab plus chemotherapy in metastatic non-small-cell lung cancer. N. Engl. J. Med.

[5] Atkins MB (2018). Safety and efficacy of axitinib (axi) in combination with pembrolizumab (pembro) in patients (pts) with advanced renal cell cancer (aRCC). J. Clin. Oncol..

[6] Lee C-H (2018). Lenvatinib + pembrolizumab in patients with renal cell carcinoma: updated results. J. Clin. Oncol..

[7] Wolchok JD (2013). Nivolumab plus ipilimumab in advanced melanoma. N. Engl. J. Med..

[8] Weber JS (2016). Sequential administration of nivolumab and ipilimumab with a planned switch in patients with advanced melanoma (CheckMate 064): an open-label, randomised, phase 2 trial. Lancet Oncol..

[9] Larkin J (2015). Combined nivolumab and ipilimumab or monotherapy in untreated melanoma. N. Engl. J. Med..

[10] Tang J, Shalabi A, Hubbard-Lucey VM (2018). Comprehensive analysis of the clinical immuno-oncology landscape. Ann. Oncol..

[11] Schmidt C (2017). The benefits of immunotherapy combinations. Nature.

[12] Ott PA, Hodi FS, Kaufman HL, Wigginton JM, Wolchok JD (2017). Combination immunotherapy: a road map. J. Immunother. Cancer.

[13] Rosenberg SA (1985). Observations on the systemic administration of autologous lymphokine-activated killer cells and recombinant interleukin-2 to patients with metastatic cancer. N. Engl. J. Med..

[14] Lotze MT (1986). High-dose recombinant interleukin 2 in the treatment of patients with disseminated cancer. Responses, treatment-related morbidity, and histologic findings. Jama.

[15] Rosenberg SA (1989). Experience with the use of high-dose interleukin-2 in the treatment of 652 cancer patients. Ann. Surg..

[16] Yang JC (2003). Randomized study of high-dose and low-dose interleukin-2 in patients with metastatic renal cancer. J. Clin. Oncol..

[17] Havel JJ, Chowell D, Chan TA (2019). The evolving landscape of biomarkers for checkpoint inhibitor immunotherapy. Nat. Rev. Cancer.

[18] Riley RS, June CH, Langer R, Mitchell MJ (2019). Delivery technologies for cancer immunotherapy. Nat. Rev. Drug Discov..

[19] Patel SJ (2017). Identification of essential genes for cancer immunotherapy. Nature.

[20] Manguso RT (2017). In vivo CRISPR screening identifies Ptpn2 as a cancer immunotherapy target. Nature.

[21] Tu, M. M. et al. Targeting DDR2 enhances tumor response to anti-PD-1 immunotherapy. *Sci. Adv.* **5**, eaav2437 (2019). 10.1126/sciadv.aav2437 PMC6382401 30801016

[22] Matsushima K, Larsen CG, DuBois GC, Oppenheim JJ (1989). Purification and characterization of a novel monocyte chemotactic and activating factor produced by a human myelomonocytic cell line. J. Exp. Med..

[23] Zernecke A, Weber C (2009). Chemokines in the vascular inflammatory response of atherosclerosis. Cardiovasc. Res..

[24] Aiello RJ (1999). Monocyte chemoattractant protein-1 accelerates atherosclerosis in apolipoprotein E-deficient mice. Arterioscler. Thromb. Vasc. Biol..

[25] Antonelli A (2009). High values of Th1 (CXCL10) and Th2 (CCL2) chemokines in patients with psoriatic arthtritis. Clin. Exp. Rheumatol..

[26] Jiang S, Wang Q, Wang Y, Song X, Zhang Y (2019). Blockade of CCL2/CCR2 signaling pathway prevents inflammatory monocyte recruitment and attenuates OVA-induced allergic asthma in mice. Immunol. Lett..

[27] Li L (2018). High levels of CCL2 or CCL4 in the tumor microenvironment predict unfavorable survival in lung adenocarcinoma. Thorac. Cancer.

[28] Ueno T (2000). Significance of macrophage chemoattractant protein-1 in macrophage recruitment, angiogenesis, and survival in human breast cancer. Clin. Cancer Res..

[29] Lebrecht A (2004). Monocyte chemoattractant protein-1 serum levels in patients with breast cancer. Tumour Biol..

[30] Li X (2017). Targeting of tumour-infiltrating macrophages via CCL2/CCR2 signalling as a therapeutic strategy against hepatocellular carcinoma. Gut.

[31] Huang B (2007). CCL2/CCR2 pathway mediates recruitment of myeloid suppressor cells to cancers. Cancer Lett..

[32] Qian BZ (2011). CCL2 recruits inflammatory monocytes to facilitate breast-tumour metastasis. Nature.

[33] Kim WJ (2010). Predictive value of progression-related gene classifier in primary non-muscle invasive bladder cancer. Mol. Cancer.

[34] Sanchez-Carbayo M, Socci ND, Lozano J, Saint F, Cordon-Cardo C (2006). Defining molecular profiles of poor outcome in patients with invasive bladder cancer using oligonucleotide microarrays. J. Clin. Oncol..

[35] Deshmane SL, Kremlev S, Amini S, Sawaya BE (2009). Monocyte chemoattractant protein-1 (MCP-1): an overview. J. Interferon Cytokine Res..

[36] Pozzobon T, Goldoni G, Viola A, Molon B (2016). CXCR4 signaling in health and disease. Immunol. Lett..

[37] Said N, Sanchez-Carbayo M, Smith SC, Theodorescu D (2012). RhoGDI2 suppresses lung metastasis in mice by reducing tumor versican expression and macrophage infiltration. J. Clin. Invest..

[38] Fridlender ZG (2010). CCL2 blockade augments cancer immunotherapy. Cancer Res..

[39] Dancik GM (2015). An online tool for evaluating diagnostic and prognostic gene expression biomarkers in bladder cancer. BMC Urol..

[40] Mirzadegan T (2000). Identification of the binding site for a novel class of CCR2b chemokine receptor antagonists: binding to a common chemokine receptor motif within the helical bundle. J. Biol. Chem..

[41] Furuichi K (2003). CCR2 signaling contributes to ischemia-reperfusion injury in kidney. J. Am. Soc. Nephrol..

[42] Kim J (2014). IL-33-induced hematopoietic stem and progenitor cell mobilization depends upon CCR2. J. Immunol..

[43] Miller RE (2012). CCR2 chemokine receptor signaling mediates pain in experimental osteoarthritis. Proc. Natl Acad. Sci. USA.

[44] Linde N (2018). Macrophages orchestrate breast cancer early dissemination and metastasis. Nat. Commun..

[45] Yang SJ, IglayReger HB, Kadouh HC, Bodary PF (2009). Inhibition of the chemokine (C-C motif) ligand 2/chemokine (C-C motif) receptor 2 pathway attenuates hyperglycaemia and inflammation in a mouse model of hepatic steatosis and lipoatrophy. Diabetologia.

[46] Lourenco S (2015). Macrophage migration inhibitory factor-CXCR4 is the dominant chemotactic axis in human mesenchymal stem cell recruitment to tumors. J. Immunol..

[47] Arendt LM (2013). Obesity promotes breast cancer by CCL2-mediated macrophage recruitment and angiogenesis. Cancer Res..

[48] Kitagawa K (2004). Blockade of CCR2 ameliorates progressive fibrosis in kidney. Am. J. Pathol..

[49] Chen S (2015). Combination of 4-1BB agonist and PD-1 antagonist promotes antitumor effector/memory CD8 T cells in a poorly immunogenic tumor model. Cancer Immunol. Res..

[50] Kleffel S (2015). Melanoma cell-intrinsic PD-1 receptor functions promote tumor growth. Cell.

[51] Juneja VR (2017). PD-L1 on tumor cells is sufficient for immune evasion in immunogenic tumors and inhibits CD8 T cell cytotoxicity. J. Exp. Med..

[52] Duan M (2012). Distinct macrophage subpopulations characterize acute infection and chronic inflammatory lung disease. J. Immunol..

[53] Janssen WJ (2011). Fas determines differential fates of resident and recruited macrophages during resolution of acute lung injury. Am. J. Respir. Crit. Care Med..

[54] Loyher PL (2018). Macrophages of distinct origins contribute to tumor development in the lung. J. Exp. Med..

[55] Bonapace L (2014). Cessation of CCL2 inhibition accelerates breast cancer metastasis by promoting angiogenesis. Nature.

[56] Casey AE, Laster WR, Ross GL (1951). Sustained enhanced growth of carcinoma EO771 in C57 black mice. Proc. Soc. Exp. Biol. Med..

[57] Robertson AG (2017). Comprehensive molecular characterization of muscle-invasive bladder cancer. Cell.

[58] Nywening TM (2016). Targeting tumour-associated macrophages with CCR2 inhibition in combination with FOLFIRINOX in patients with borderline resectable and locally advanced pancreatic cancer: a single-centre, open-label, dose-finding, non-randomised, phase 1b trial. Lancet Oncol..

[59] Linehan D (2018). Overall survival in a trial of orally administered CCR2 inhibitor CCX872 in locally advanced/metastatic pancreatic cancer: Correlation with blood monocyte counts. J. Clin. Oncol..

[60] Sandhu SK (2013). A first-in-human, first-in-class, phase I study of carlumab (CNTO 888), a human monoclonal antibody against CC-chemokine ligand 2 in patients with solid tumors. Cancer Chemother. Pharm..

[61] Pienta KJ (2013). Phase 2 study of carlumab (CNTO 888), a human monoclonal antibody against CC-chemokine ligand 2 (CCL2), in metastatic castration-resistant prostate cancer. Invest. N. Drugs.

[62] Redman JM, Hill EM, AlDeghaither D, Weiner LM (2015). Mechanisms of action of therapeutic antibodies for cancer. Mol. Immunol..

[63] Andrews LP, Marciscano AE, Drake CG, Vignali DA (2017). LAG3 (CD223) as a cancer immunotherapy target. Immunol. Rev..

[64] Woo SR (2012). Immune inhibitory molecules LAG-3 and PD-1 synergistically regulate T-cell function to promote tumoral immune escape. Cancer Res..

[65] Huang RY (2015). LAG3 and PD1 co-inhibitory molecules collaborate to limit CD8+ T cell signaling and dampen antitumor immunity in a murine ovarian cancer model. Oncotarget.

[66] Beltra JC (2020). Developmental relationships of four exhausted CD8(+) T cell subsets reveals underlying transcriptional and epigenetic landscape control mechanisms. Immunity.

[67] Mukaida, N., Nosaka, T., Nakamoto, Y. & Baba, T. Lung macrophages: multifunctional regulator cells for metastatic cells. *Int. J. Mol. Sci.*10.3390/ijms20010116 (2018). 10.3390/ijms20010116 PMC6337639 30597969

[68] Yu YR (2016). Flow cytometric analysis of myeloid cells in human blood, bronchoalveolar lavage, and lung tissues. Am. J. Respir. Cell Mol. Biol..

[69] Hussell T, Bell TJ (2014). Alveolar macrophages: plasticity in a tissue-specific context. Nat. Rev. Immunol..

[70] McQuattie-Pimentel AC, Budinger GRS, Ballinger MN (2018). Monocyte-derived alveolar macrophages: the dark side of lung repair?. Am. J. Respir. Cell Mol. Biol..

[71] Lin, C. H., Yeh, Y. C. & Yang, K. D. Functions and therapeutic targets of Siglec-mediated infections, inflammations and cancers. *J. Formos Med. Assoc*. 10.1016/j.jfma.2019.10.019 (2019). 10.1016/j.jfma.2019.10.019 31882261

[72] Fraschilla I, Pillai S (2017). Viewing Siglecs through the lens of tumor immunology. Immunol. Rev..

[73] Komohara Y, Ohnishi K, Takeya M (2017). Possible functions of CD169-positive sinus macrophages in lymph nodes in anti-tumor immune responses. Cancer Sci..

[74] Laubli H (2014). Engagement of myelomonocytic Siglecs by tumor-associated ligands modulates the innate immune response to cancer. Proc. Natl Acad. Sci. USA.

[75] Poczobutt JM (2016). Expression profiling of macrophages reveals multiple populations with distinct biological roles in an immunocompetent orthotopic model of lung cancer. J. Immunol..

[76] Higgs BW (2018). Interferon gamma messenger RNA signature in tumor biopsies predicts outcomes in patients with non-small cell lung carcinoma or urothelial cancer treated with Durvalumab. Clin. Cancer Res.

[77] Karachaliou N (2018). Interferon gamma, an important marker of response to immune checkpoint blockade in non-small cell lung cancer and melanoma patients. Ther. Adv. Med. Oncol..

[78] Tu MM (2019). Targeting DDR2 enhances tumor response to anti-PD-1 immunotherapy. Sci. Adv..

[79] Atif SM, Gibbings SL, Jakubzick CV (2018). Isolation and identification of interstitial macrophages from the lungs using different digestion enzymes and staining strategies. Methods Mol. Biol..

[80] McGettigan B (2019). Dietary lipids differentially shape nonalcoholic steatohepatitis progression and the transcriptome of Kupffer cells and infiltrating macrophages. Hepatology.

[81] Gibbings SL, Jakubzick CV (2018). Isolation and characterization of mononuclear phagocytes in the mouse lung and lymph nodes. Methods Mol. Biol..

[82] Gautier EL (2012). Gene-expression profiles and transcriptional regulatory pathways that underlie the identity and diversity of mouse tissue macrophages. Nat. Immunol..

[83] Li B, Dewey CN (2011). RSEM: accurate transcript quantification from RNA-Seq data with or without a reference genome. BMC Bioinforma..

[84] Langmead B, Salzberg SL (2012). Fast gapped-read alignment with Bowtie 2. Nat. Methods.

[85] Law CW, Chen Y, Shi W, Smyth GK (2014). voom: Precision weights unlock linear model analysis tools for RNA-seq read counts. Genome Biol..

[86] Barrett T (2013). NCBI GEO: archive for functional genomics data sets–update. Nucleic Acids Res..

[87] Dyrskjot L (2004). Gene expression in the urinary bladder: a common carcinoma in situ gene expression signature exists disregarding histopathological classification. Cancer Res..

[88] Dyrskjot L (2007). Gene expression signatures predict outcome in non-muscle-invasive bladder carcinoma: a multicenter validation study. Clin. Cancer Res..

[89] Riester M (2012). Combination of a novel gene expression signature with a clinical nomogram improves the prediction of survival in high-risk bladder cancer. Clin. Cancer Res..

[90] Lindgren D (2010). Combined gene expression and genomic profiling define two intrinsic molecular subtypes of urothelial carcinoma and gene signatures for molecular grading and outcome. Cancer Res..

[91] Lindgren D (2012). Integrated genomic and gene expression profiling identifies two major genomic circuits in urothelial carcinoma. PLoS ONE.

[92] Choi W (2014). Identification of distinct basal and luminal subtypes of muscle-invasive bladder cancer with different sensitivities to frontline chemotherapy. Cancer Cell.

[93] Smith SC, Baras AS, Owens CR, Dancik G, Theodorescu D (2012). Transcriptional signatures of Ral GTPase are associated with aggressive clinicopathologic characteristics in human cancer. Cancer Res..

[94] Kolesnikov N (2015). ArrayExpress update–simplifying data submissions. Nucleic Acids Res..

[95] Stransky N (2006). Regional copy number-independent deregulation of transcription in cancer. Nat. Genet..

[96] Blaveri E (2005). Bladder cancer outcome and subtype classification by gene expression. Clin. Cancer Res..

[97] Miller JA (2011). Strategies for aggregating gene expression data: the collapseRows R function. BMC Bioinforma..

[98] Newman AM (2015). Robust enumeration of cell subsets from tissue expression profiles. Nat. Methods.

[99] Charoentong P (2017). Pan-cancer immunogenomic analyses reveal genotype-immunophenotype relationships and predictors of response to checkpoint blockade. Cell Rep..

