# Supplementary material for: Inhibition of the CCL2 receptor, CCR2, enhances tumor response to immune checkpoint therapy
Source: Commun Biol. 2020 Nov 27;3:720. doi: 10.1038/s42003-020-01441-y (PMC7699641; doi:10.1038/s42003-020-01441-y)
Supplement: Supplementary file 1 — Supplementary Information [file 42003_2020_1441_MOESM1_ESM.pdf]

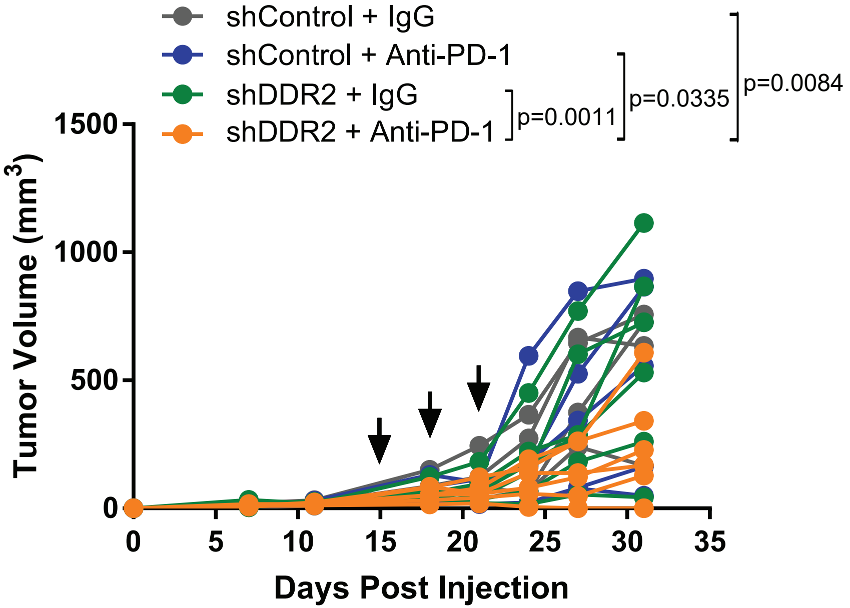

Supplementary Figure 1. Combination therapy targeting DDR2 with anti-PD-1 treatment for *in vivo* NA13 tumors. Subcutaneous tumour growth in syngeneic mice receiving NA13 shDDR2 #1 cells stably expressing shControl (n=5 biologically independent mice per group) or shDDR2 (n=6 biologically independent mice per group) Each dot/line represents a single mouse. Arrows indicate days of IgG or anti-PD-1 treatment.

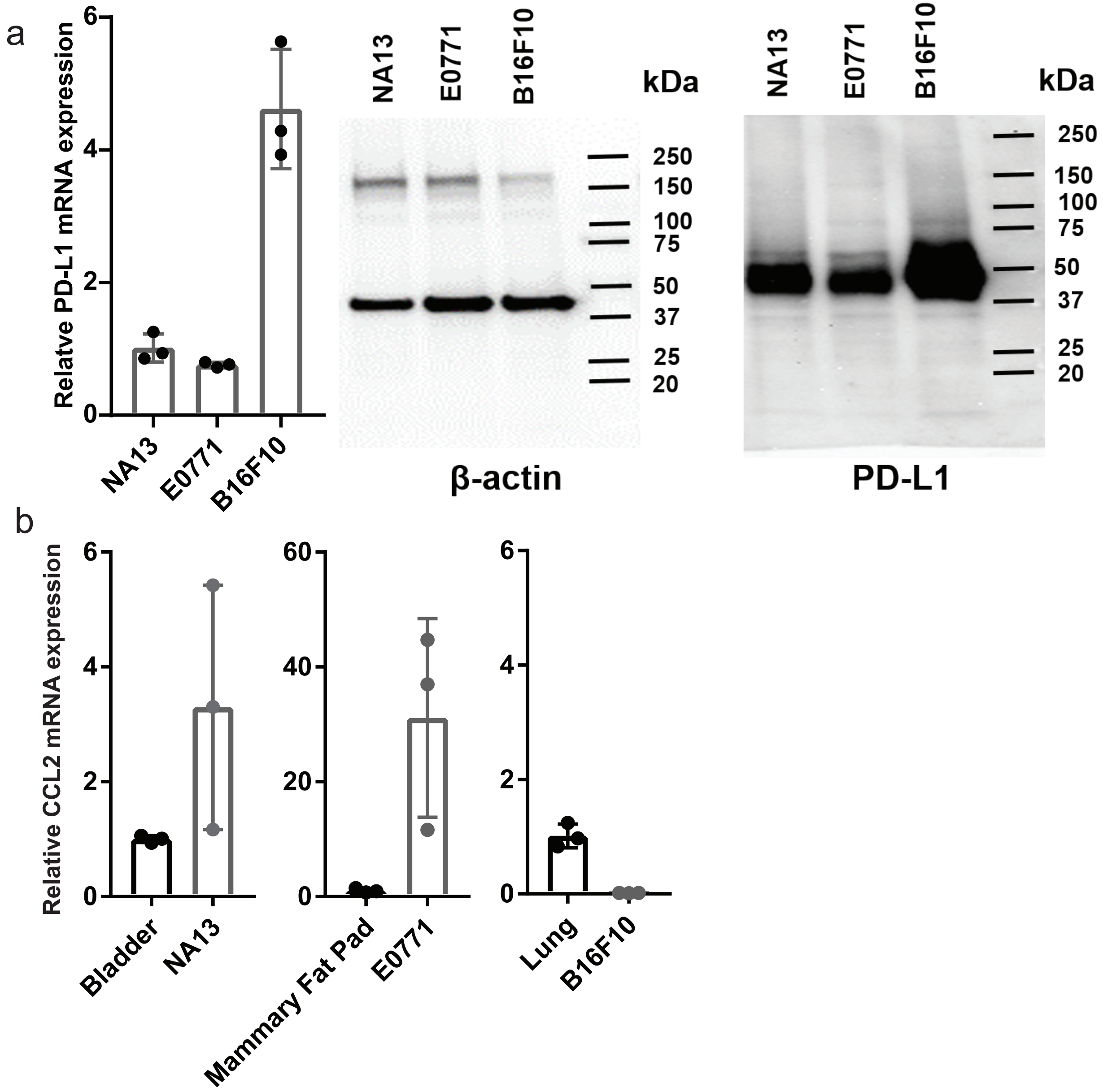

Supplementary Figure 2. Analysis of PD-L1 and CCL2 expression levels. (a) qPCR and immunoblot analysis of PD-L1. **(b)** CCL2 expression levels in NA13, B16F10 and E0771 cell lines. Mean +/- SD. n=3 independent samples per cell line.

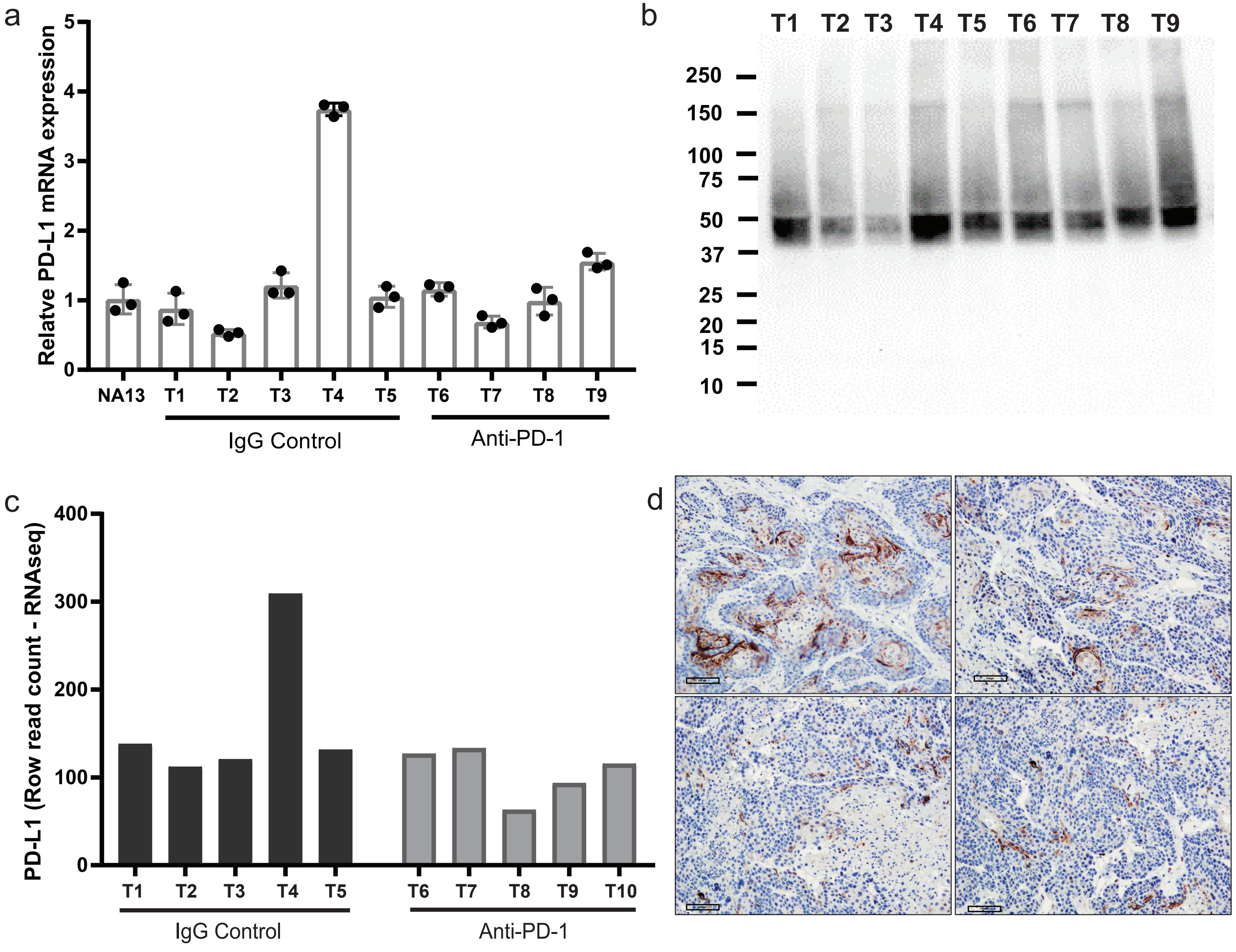

Supplementary Figure 3. PD-L1 expression levels in in vivo grown NA13 tumors. **(a)** qPCR, **(b)** immunoblot and **(c)** RNAseq analysis of PD-L1 expression in *in vitro* grown NA13 (NA13) and *in vivo* grown NA13 tumors in syngeneic mice (T1, T2, T3, T4, T5, T6, T7, T8 and T9). **(d)** Immunohistochemistry for PD-L1 expression (brown) in formalin-fixed and paraffin-embedded NA13 tumor sections harvested 25 days after injection. Scale bar represents represents 100um.

.

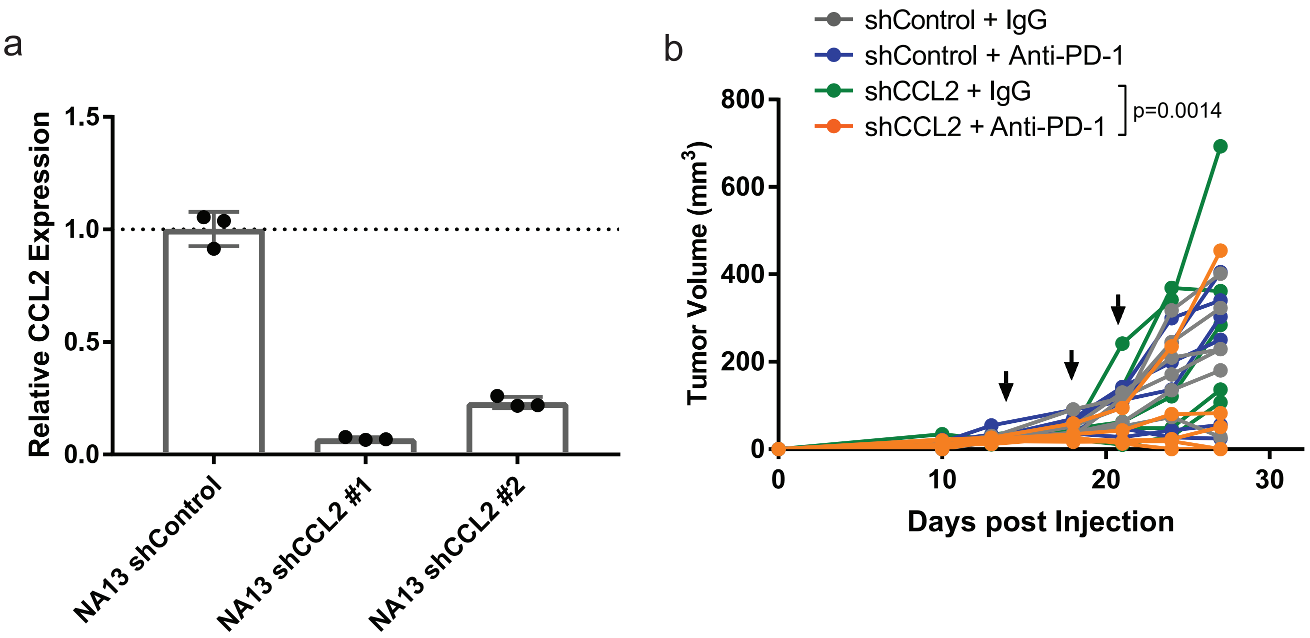

Supplemental Figure 4. shRNA-mediated knockdown of CCL2 expression. (a) qPCR analysis of NA13 shControl and shCCL2 #1 and #2. n=3 independent samples per cell line. Mean +/- SD **(b)** Subcutaneous tumor growth in syngeneic mice receiving NA13 cells stably expressing shControl (n=6 biologically independent mice) or shRNA-mediated knockdown of CCL2 (shCCL2) (n = 5 biologically independent mice). Data representative of two independent experiments. Statistical significance was determined by two-way ANOVA. Arrows indicate dates in which IgG or anti-PD-1 treatment were given.

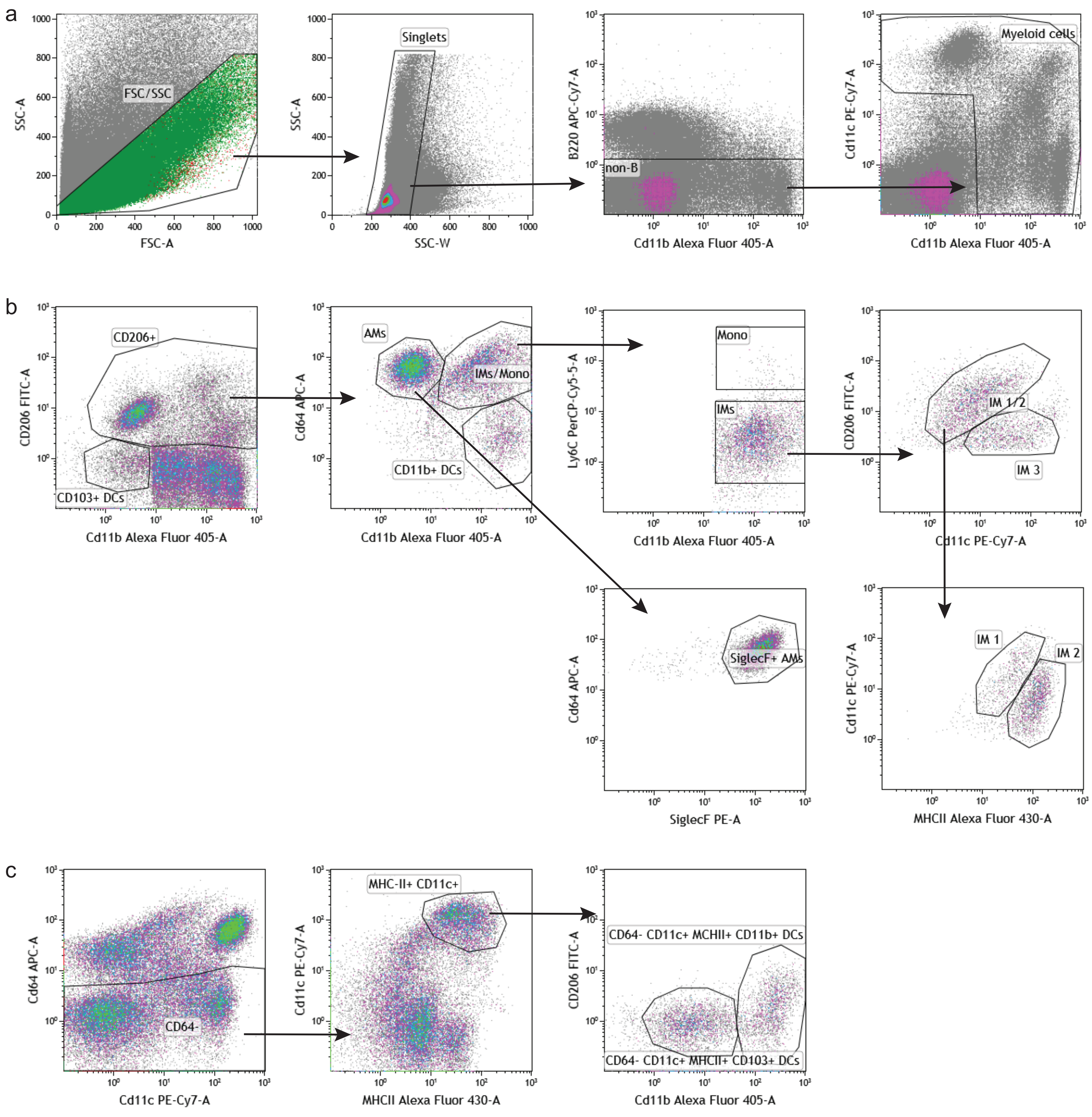

Supplementary Figure 5. Representative gating of flow cytometry data for lungs from B16F10-tumor bearing mice. (a) Myeloid cells, **(b)** and **(c)** dendritic cells (DCs), alveolar macrophages (AM), interstitial macrophages (IM), monocytes (Mono).

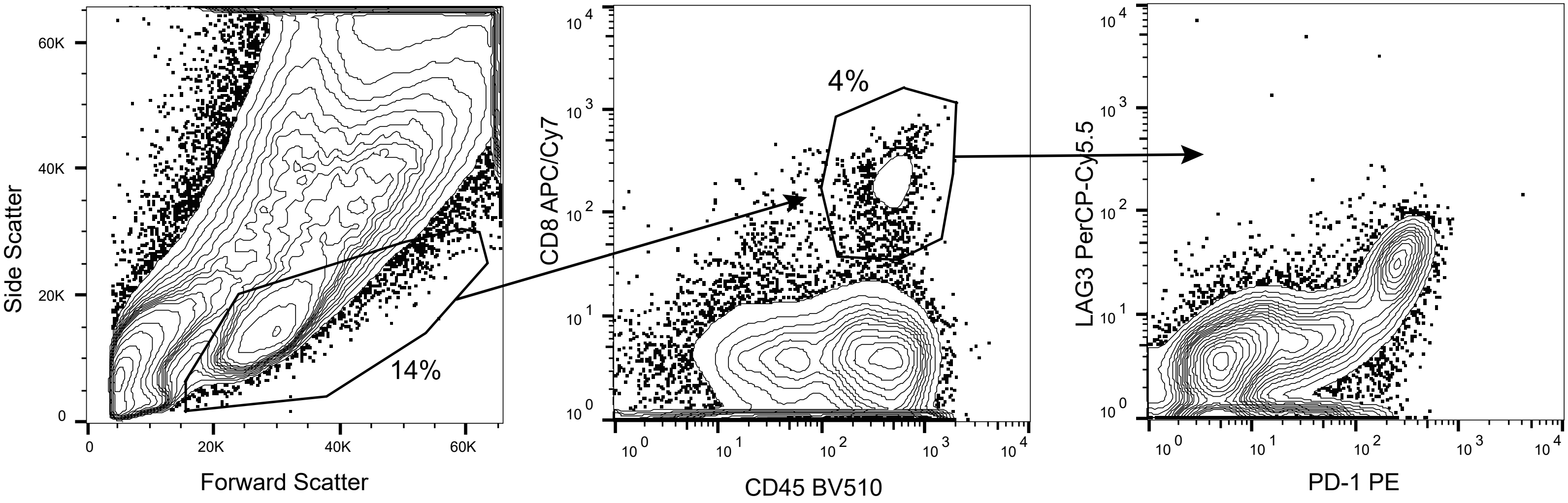

Supplementary Figure 6. Representative gating of flow cytometry data for E0771 tumors.

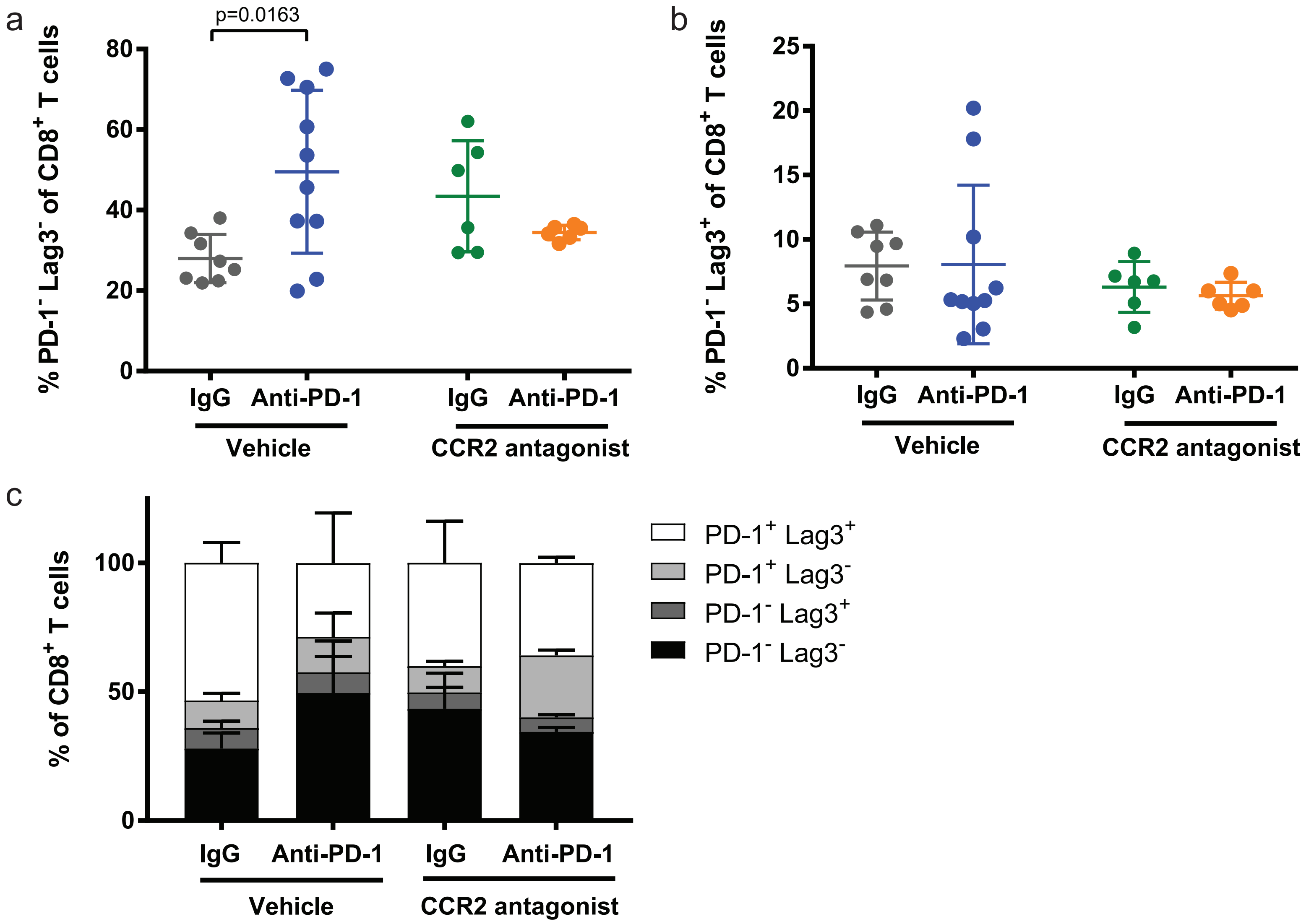

Supplementary Figure 7. Tumor-infiltrating CD8 T cell subsets defined by PD-1 and LAG3 expression. PD-1 and LAG3 expression. Percentage of **(a)** PD-1- LAG3- and **(b)** PD-1- LAG3+ of CD8+ T cells. (**c**) Stacked graph depicting the four CD8 subsets according to PD-1/LAG3 expression. Mean ± SD. Statistical significance determined using two-way ANOVA. If statistical significance is not indicated in the figure, $p > 0.05$ and not statistically significant. Each dot represents a biologically independent mouse.

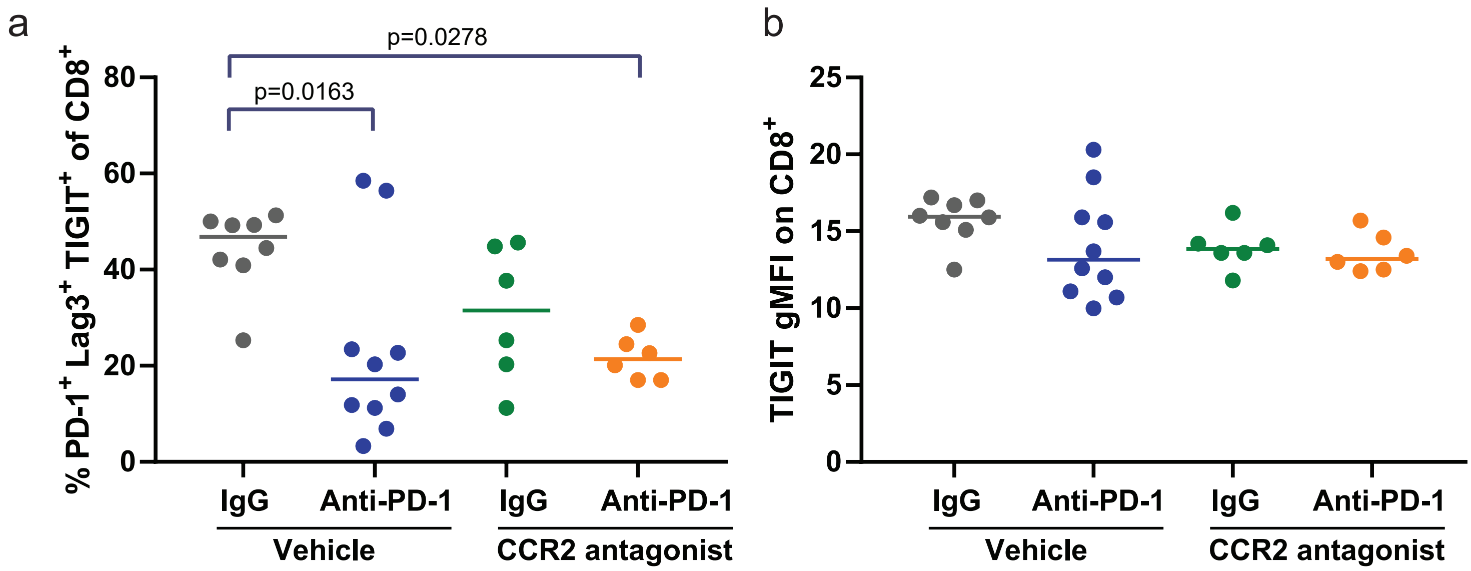

Supplementary Figure 8. TIGIT expression on tumor-infiltrating CD8 T cells. Flow cytometry-based analysis of **(a)** frequency of PD-1+ Lag3+ TIGIT+ CD8 T cells and **(b)** expression levels of TIGIT. Each dot represents a biologically independent mouse. Horizontal line represents mean. Statistical significance determined using one-way ANOVA.

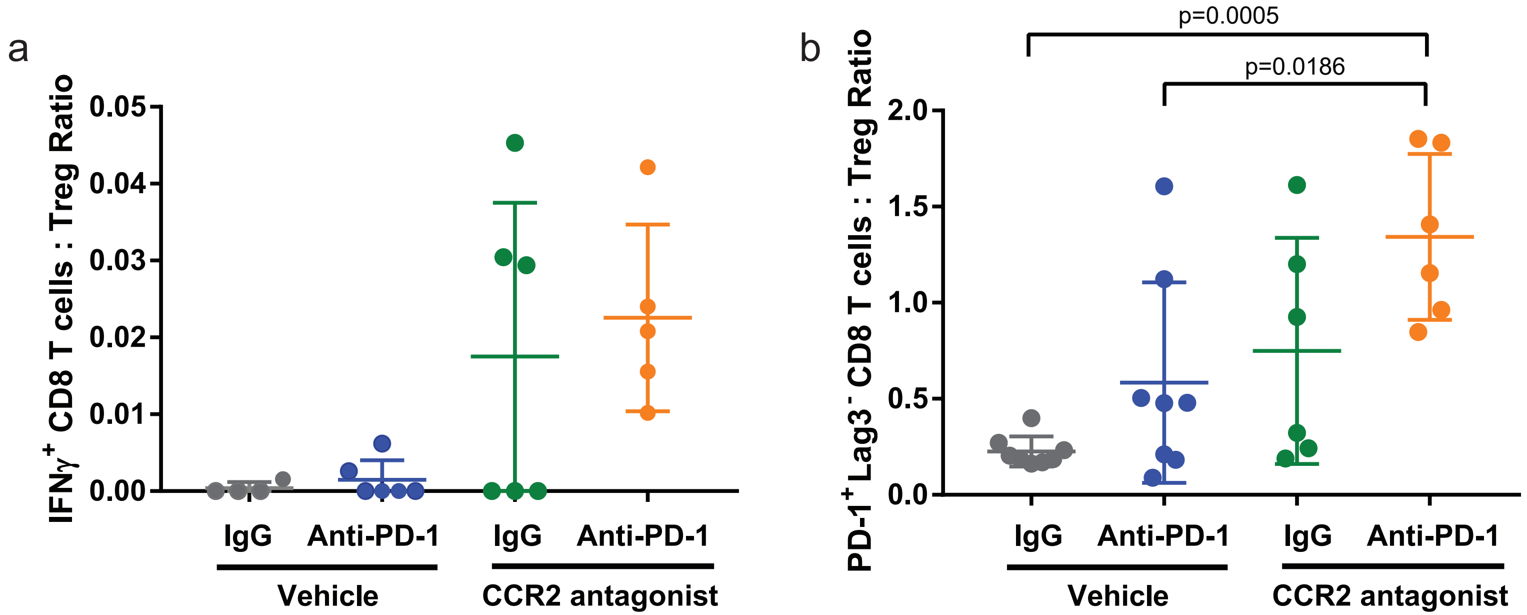

Supplementary Figure 9. Relative ratio of tumor-infiltrating CD8 T cells to Treg. (**a**) IFNγ+ CD8+ T cells relative to Treg. (**b**) PD-1+ Lag3- CD8+ T cells relative to Treg. Mean ± SD. Statistical significance determined by one-way ANOVA. If statistical significance is not indicated in the figure, $p > 0.05$. Each dot represents a biologically independent mouse.

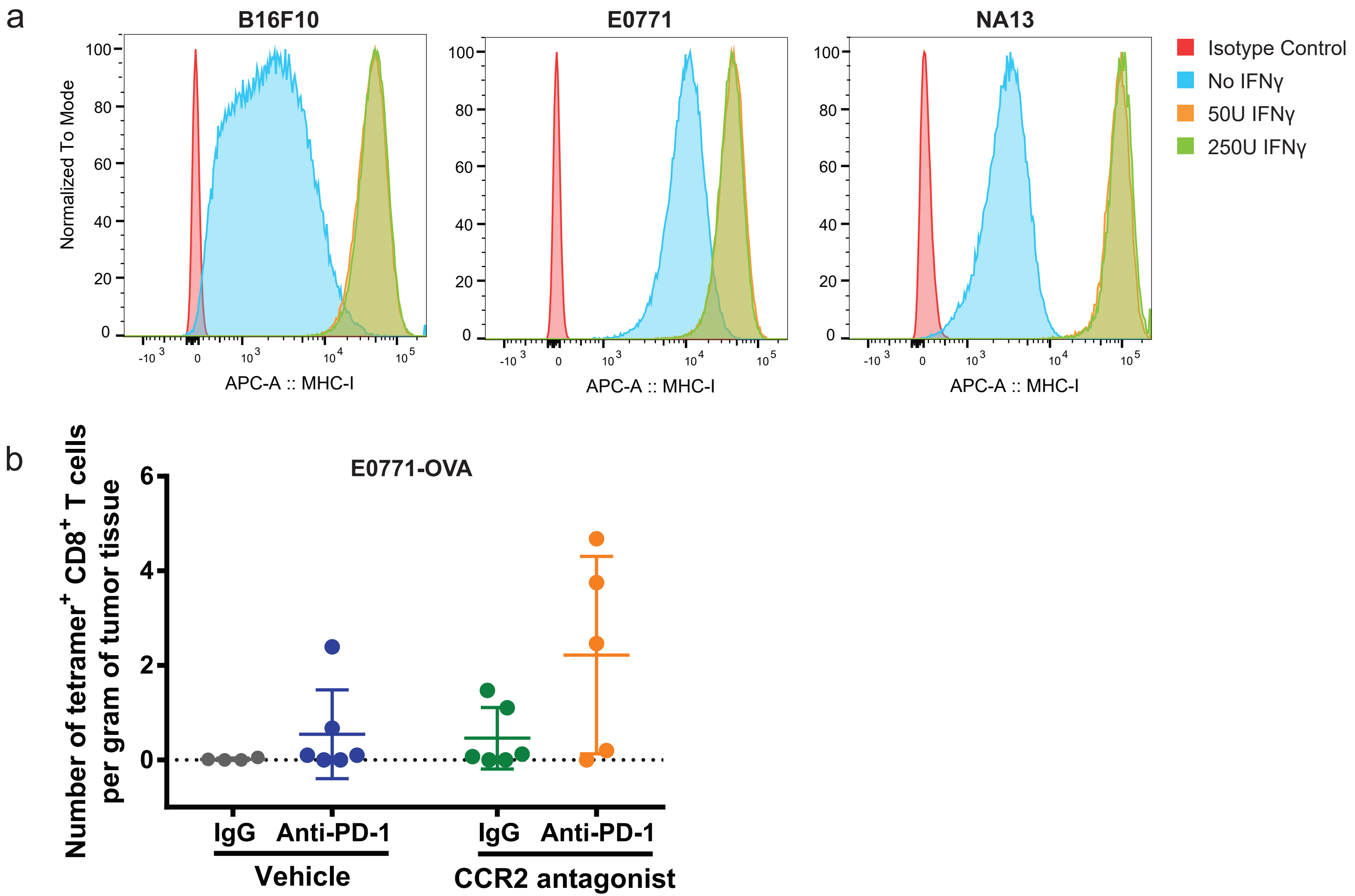

Supplementary Figure 10. CD8 T cell recognition of tumor cells (a) Flow cytometry-based analysis of MHC-I expression levels by B16F10, E0771 and NA13 tumor cells. **(b)** Localization of peptide-specific CD8 T cells in E0771 subcutaneous tumors grown in syngeneic mice. Each dot represents a biologically independent mouse. No statistical significance as determined by two-wayANOVA. Mean ± SEM

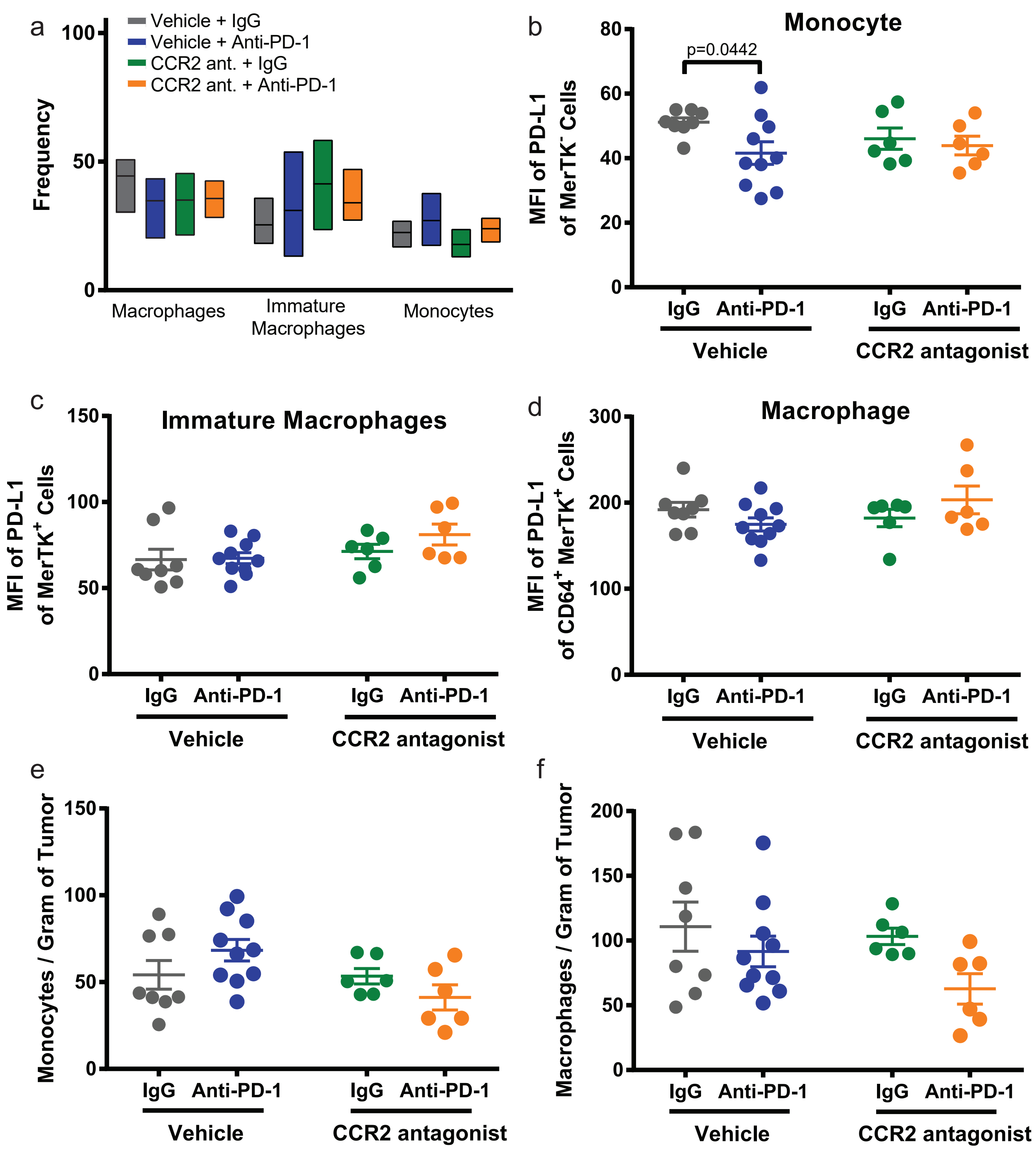

Supplementary Figure 11. Tumor-infiltrating myeloid cell populations in E0771 tumors. (a) Proportion of monocytes, immature macrophages and macrophages of the myeloid population present in the tumor. **(b-d)** Mean fluorescence intensity of PD-L1 expression on monocytes, immature macrophages and macrophages. **(e-f)** Monocytes and macrophages per gram of bulk E0771 tumor. Each dot represents a biologically independent mouse. Mean +/- SEM. Statistical significance determined by two-way ANOVA.

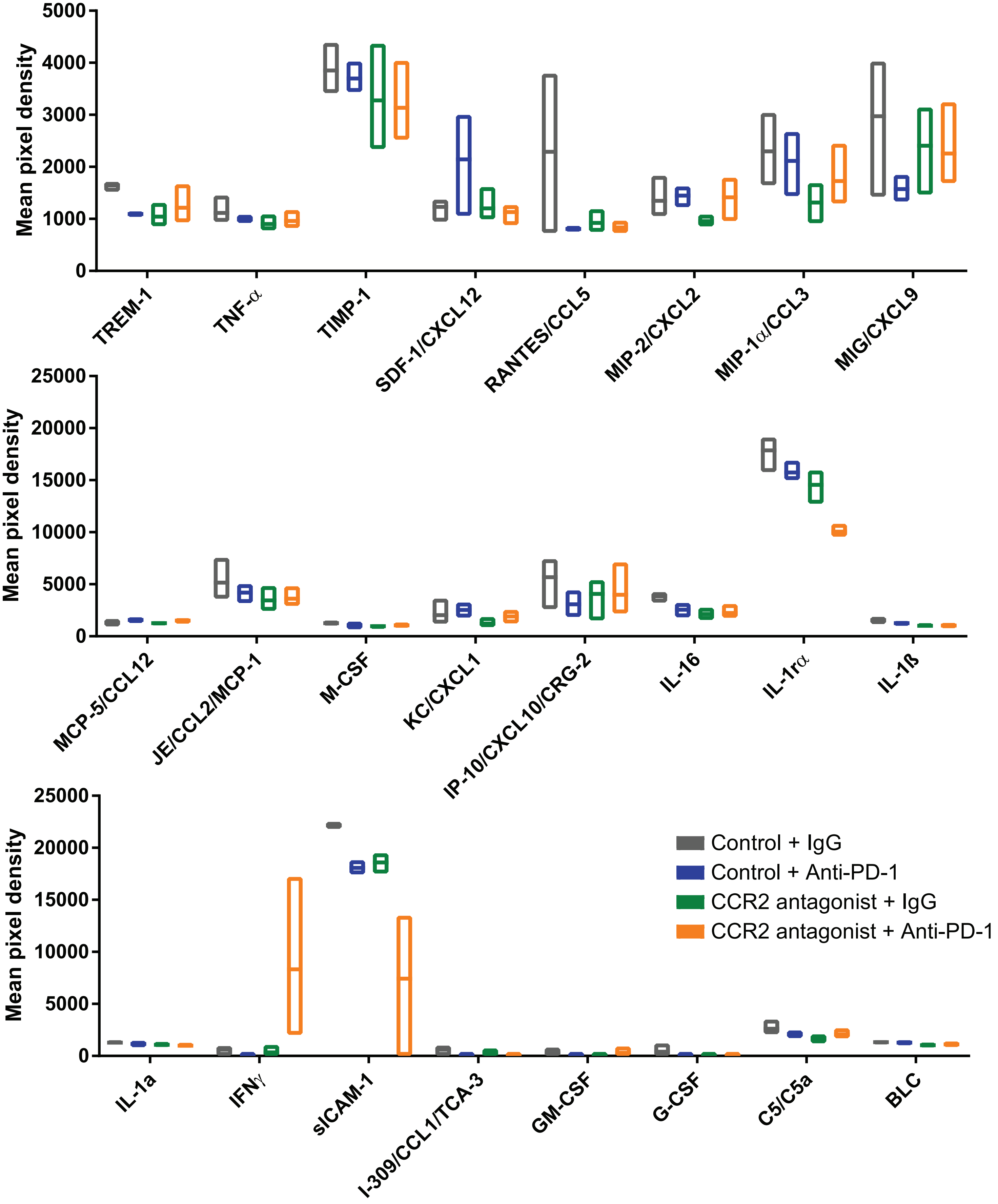

Supplementary Figure 12. Cytokine panel analysis. Cytokine array of tumor tissue harvested 25 days post subcutaneous E0771 injection into mice. Data is presented as mean pixel density as determined by chemiluminescence measured with Biorad ChemiDoc MP Imaging system. Mean +/- min/max. n=3 biologically independent mice per treatment group. Statistically significant data is present in Fig. 5e.

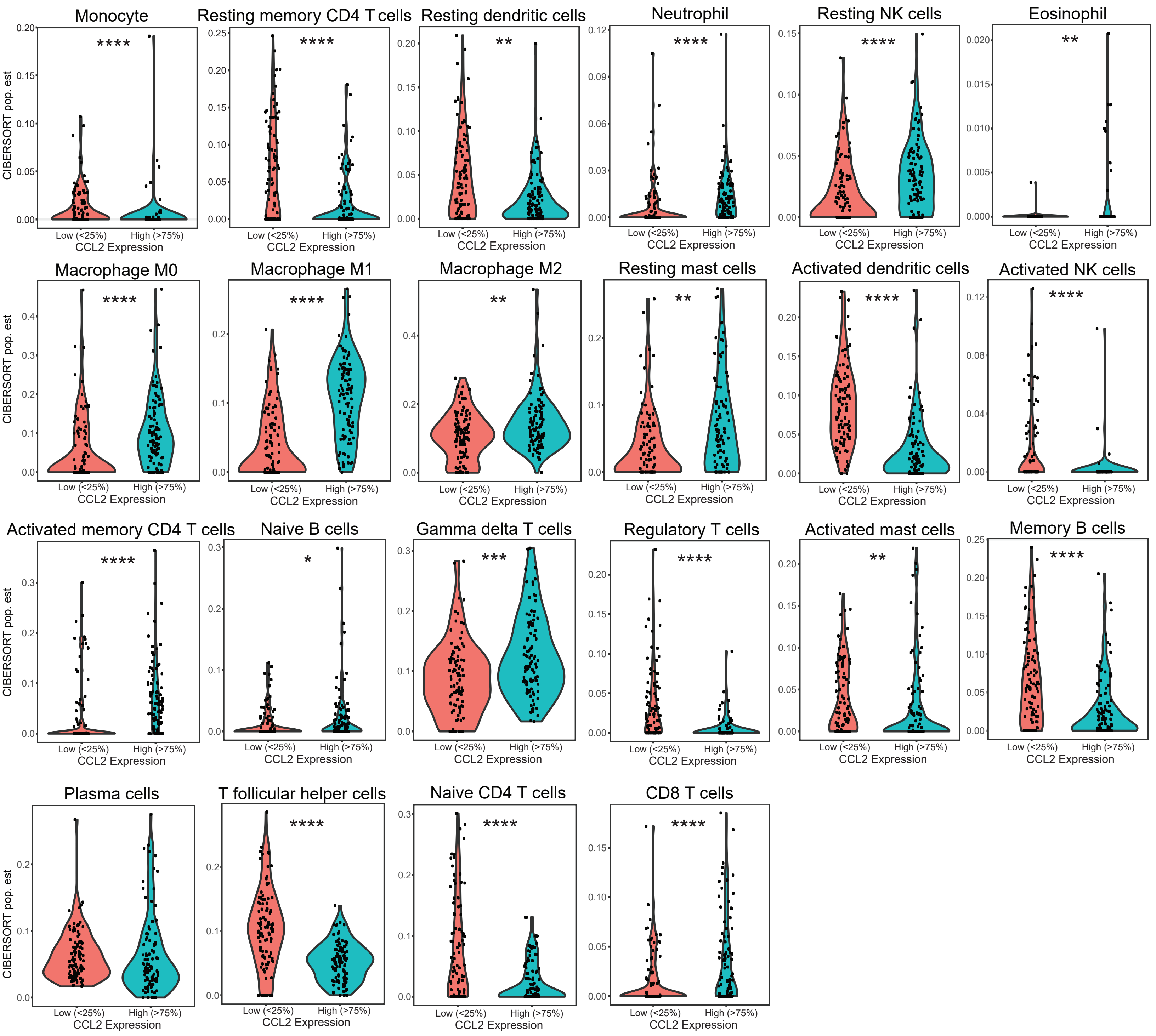

Supplementary Figure 13. CIBERSORT analysis of bladder cancer datasets from The Cancer Genome Atlas (TCGA) Relative abundance of tumor-infiltrating immune cell populations determined by the CIBERSORT methodology in bladder cancer patients from RNA-seq data in TCGA as a function of CCL2 expression. *$p < 0.05$, **$p < 0.001$,,***$p < 0.001$, ****$p < 0.0001$.
