# Supplementary material for: Inhibition of the CCL2 receptor, CCR2, enhances tumor response to immune checkpoint therapy
Source: Commun Biol. 2020 Nov 27;3:720. doi: 10.1038/s42003-020-01441-y (PMC7699641; doi:10.1038/s42003-020-01441-y)
Supplement: Supplementary file 2 — Description of Additional Supplementary File [file 42003_2020_1441_MOESM2_ESM.pdf]

Description of Additional Supplementary File

File name: Supplementary Data 1

Description: Source data for the main and supplementary figures
