# Supplementary material for: Inhibition of the CCL2 receptor, CCR2, enhances tumor response to immune checkpoint therapy
Source: Commun Biol. 2020 Nov 27;3:720. doi: 10.1038/s42003-020-01441-y (PMC7699641; doi:10.1038/s42003-020-01441-y)
Supplement: Supplementary file 4 — Reporting Summary [file 42003_2020_1441_MOESM4_ESM.pdf]

nature research

Corresponding author(s): Dan Theodorescu

Last updated by author(s): Oct 17, 2020

Reporting Summary

Nature Research wishes to improve the reproducibility of the work that we publish. This form provides structure for consistency and transparency in reporting. For further information on Nature Research policies, see our Editorial Policies and the Editorial Policy Checklist.

Statistics

For all statistical analyses, confirm that the following items are present in the figure legend, table legend, main text, or Methods section.

n/a	Confirmed	
☐	☒	The exact sample size (n) for each experimental group/condition, given as a discrete number and unit of measurement
☐	☒	A statement on whether measurements were taken from distinct samples or whether the same sample was measured repeatedly
☐	☒	The statistical test(s) used AND whether they are one- or two-sided<br>*Only common tests should be described solely by name; describe more complex techniques in the Methods section.*
☐	☒	A description of all covariates tested
☐	☒	A description of any assumptions or corrections, such as tests of normality and adjustment for multiple comparisons
☐	☒	A full description of the statistical parameters including central tendency (e.g. means) or other basic estimates (e.g. regression coefficient) AND variation (e.g. standard deviation) or associated estimates of uncertainty (e.g. confidence intervals)
☐	☒	For null hypothesis testing, the test statistic (e.g. F, t, r) with confidence intervals, effect sizes, degrees of freedom and P value noted<br>*Give P values as exact values whenever suitable.*
☒	☐	For Bayesian analysis, information on the choice of priors and Markov chain Monte Carlo settings
☒	☐	For hierarchical and complex designs, identification of the appropriate level for tests and full reporting of outcomes
☒	☐	Estimates of effect sizes (e.g. Cohen's d, Pearson's r), indicating how they were calculated

Our web collection on statistics for biologists contains articles on many of the points above.

Software and code

Policy information about availability of computer code

Data collection	Flow cytometry: CyAn ADP flow cytometer. Imaging: Olympus BX43 Light microscope, Olympus DP26 Digital Camera, iBright imaging system, Biorad ChemiDoc MP Imaging system. QuantStudio™ 6 Flex Real-Time PCR System. Sequencing: Illumina HiSeq2500, Nextseq500, Novaseq 6000 (https://www.illumina.com/) . For CIBERSORT analysis, data was downloaded directly from the The Cancer Immune Atlas (TCIA) website. For CCL2 expression analysis, processed data was downloaded from the Gene Expression Omnibus (AUH-1 cohort: Accession #GSE3167; AUH-2: GSE5479; CNUH, GSE13507; DFCI: GSE31684; Lindgren: GSE19915; Lindgren-2: GSE32548; MDA-1: GSE48276 and MDA-2: GSE48075; UVA: GSE37317, from Array Express, (Stransky-1 and Stransky-2: E-TABM-147or as Supplemental Material to Blaveri and MSKCC.
Data analysis	Flowjo version 10.5, GraphPad Prism 7.0, Microsoft Excel, ImageJ.RNAseq analysis: Transcript quantification was done using RSEM (v1.2.31) with default parameters and Bowtie2 (v2.1.0) as the read aligner. Reads were mapped directly to mouse transcripts and summarized at the gene level using annotations from Ensembl r91, genome build GRCm38.p5. Differential expression was performed using voom function in the limma R package. Genes with an average expected count less than 5 were removed, normalization factors were calculated, and comparisons between groups were made using the voom function using default parameters. CIBERSORT using TCGA bladder cancer gene expression was processed according to The Cancer Immune Atlas (TCIA) and the associated data as downloaded directly from the TCIA website. Preliminary analysis using the Bladder Cancer Biomarker Evaluation Tool (BC-BET) was used to evaluate the association of CCL2 expression with bladder cancer characteristics. The 12 patient cohorts included in BC-BET were then downloaded as previously described by Dancik et al., BMC urology 2019; 15(59).

For manuscripts utilizing custom algorithms or software that are central to the research but not yet described in published literature, software must be made available to editors and reviewers. We strongly encourage code deposition in a community repository (e.g. GitHub). See the Nature Research guidelines for submitting code & software for further information.

Data

Policy information about availability of data

All manuscripts must include a data availability statement. This statement should provide the following information, where applicable:

- Accession codes, unique identifiers, or web links for publicly available datasets
- A list of figures that have associated raw data
- A description of any restrictions on data availability

The authors declare that the data supporting the findings of this study are available within the paper, Supplementary Information, Source data tables or will be available upon request. There is no restriction on data availability.

Field-specific reporting

Please select the one below that is the best fit for your research. If you are not sure, read the appropriate sections before making your selection.

☒ Life sciences ☐ Behavioural & social sciences ☐ Ecological, evolutionary & environmental sciences

For a reference copy of the document with all sections, see nature.com/documents/nr-reporting-summary-flat.pdf

Life sciences study design

All studies must disclose on these points even when the disclosure is negative.

Sample size	Sample size was chosen based on a previously published studies of similar models (Said et al., The Journal of Clinical Investigation 2012;122: 1503-1518; Tu et al., Science Advances 2019;5(2).
Data exclusions	All samples that met adequate experimental conditions were included in the analysis. Samples that failed the Grubb's test for outliers in GraphPad 7.0 were excluded.
Replication	Experiments were successfully performed a minimum of two times and/or with sufficient animals per group to demonstrate statistical significance.
Randomization	Mice were randomized to treatment groups and blinded to researcher for the duration of the experiment. The same batch and number of cancer cells were injected per experiment, and all animals were housed under the same conditions
Blinding	Investigators were blinded to group allocation during the experiment. All analysis was performed consistently during all studies and all tumor counts were performed by the same investigator.

Behavioural & social sciences study design

All studies must disclose on these points even when the disclosure is negative.

Study description	*Briefly describe the study type including whether data are quantitative, qualitative, or mixed-methods (e.g. qualitative cross-sectional, quantitative experimental, mixed-methods case study).*
Research sample	*State the research sample (e.g. Harvard university undergraduates, villagers in rural India) and provide relevant demographic information (e.g. age, sex) and indicate whether the sample is representative. Provide a rationale for the study sample chosen. For studies involving existing datasets, please describe the dataset and source.*
Sampling strategy	*Describe the sampling procedure (e.g. random, snowball, stratified, convenience). Describe the statistical methods that were used to predetermine sample size OR if no sample-size calculation was performed, describe how sample sizes were chosen and provide a rationale for why these sample sizes are sufficient. For qualitative data, please indicate whether data saturation was considered, and what criteria were used to decide that no further sampling was needed.*
Data collection	*Provide details about the data collection procedure, including the instruments or devices used to record the data (e.g. pen and paper, computer, eye tracker, video or audio equipment) whether anyone was present besides the participant(s) and the researcher, and whether the researcher was blind to experimental condition and/or the study hypothesis during data collection.*
Timing	*Indicate the start and stop dates of data collection. If there is a gap between collection periods, state the dates for each sample cohort.*
Data exclusions	*If no data were excluded from the analyses, state so OR if data were excluded, provide the exact number of exclusions and the rationale behind them, indicating whether exclusion criteria were pre-established.*
Non-participation	*State how many participants dropped out/declined participation and the reason(s) given OR provide response rate OR state that no participants dropped out/declined participation.*

Randomization	*If participants were not allocated into experimental groups, state so OR describe how participants were allocated to groups, and if allocation was not random, describe how covariates were controlled.*

Ecological, evolutionary & environmental sciences study design

All studies must disclose on these points even when the disclosure is negative.

Study description	*Briefly describe the study. For quantitative data include treatment factors and interactions, design structure (e.g. factorial, nested, hierarchical), nature and number of experimental units and replicates.*
Research sample	*Describe the research sample (e.g. a group of tagged Passer domesticus, all Stenocereus thurberi within Organ Pipe Cactus National Monument), and provide a rationale for the sample choice. When relevant, describe the organism taxa, source, sex, age range and any manipulations. State what population the sample is meant to represent when applicable. For studies involving existing datasets, describe the data and its source.*
Sampling strategy	*Note the sampling procedure. Describe the statistical methods that were used to predetermine sample size OR if no sample-size calculation was performed, describe how sample sizes were chosen and provide a rationale for why these sample sizes are sufficient.*
Data collection	*Describe the data collection procedure, including who recorded the data and how.*
Timing and spatial scale	*Indicate the start and stop dates of data collection, noting the frequency and periodicity of sampling and providing a rationale for these choices. If there is a gap between collection periods, state the dates for each sample cohort. Specify the spatial scale from which the data are taken*
Data exclusions	*If no data were excluded from the analyses, state so OR if data were excluded, describe the exclusions and the rationale behind them, indicating whether exclusion criteria were pre-established.*
Reproducibility	*Describe the measures taken to verify the reproducibility of experimental findings. For each experiment, note whether any attempts to repeat the experiment failed OR state that all attempts to repeat the experiment were successful.*
Randomization	*Describe how samples/organisms/participants were allocated into groups. If allocation was not random, describe how covariates were controlled. If this is not relevant to your study, explain why.*
Blinding	*Describe the extent of blinding used during data acquisition and analysis. If blinding was not possible, describe why OR explain why blinding was not relevant to your study.*

Did the study involve field work? ☐ Yes ☐ No

Field work, collection and transport

Field conditions	*Describe the study conditions for field work, providing relevant parameters (e.g. temperature, rainfall).*
Location	*State the location of the sampling or experiment, providing relevant parameters (e.g. latitude and longitude, elevation, water depth).*
Access & import/export	*Describe the efforts you have made to access habitats and to collect and import/export your samples in a responsible manner and in compliance with local, national and international laws, noting any permits that were obtained (give the name of the issuing authority, the date of issue, and any identifying information).*
Disturbance	*Describe any disturbance caused by the study and how it was minimized.*

Reporting for specific materials, systems and methods

We require information from authors about some types of materials, experimental systems and methods used in many studies. Here, indicate whether each material, system or method listed is relevant to your study. If you are not sure if a list item applies to your research, read the appropriate section before selecting a response.

Materials & experimental systems

n/a	Involved in the study	
☐	☒	Antibodies
☐	☒	Eukaryotic cell lines
☒	☐	Palaeontology and archaeology
☐	☒	Animals and other organisms
☒	☐	Human research participants
☒	☐	Clinical data
☒	☐	Dual use research of concern

Methods

n/a	Involved in the study	
☒	☐	ChIP-seq
☐	☒	Flow cytometry
☒	☐	MRI-based neuroimaging

Antibodies

Antibodies used

Antibodies used for western blots: PD-L1 (MAB90781, R&D Systems, USA) or anti–β-actin (13E5, Cell Signaling, USA) Rabbit monoclonal antibodies Rabbit anti-mouse antibody (D5V3B, Cell Signaling Technology, USA) at a dilution of 1:100.
In Vivo experiments: Mouse anti-PD-1 antibody (IgG1-D265A) and isotype control (IgG1, clone 4F7)
Flow cytometry antibodies: APC anti-mouse H-2Db and APC mouse IgG2a K isotype control (FC) antibodies. For the CD8 T cell panel, cells were stained with CD8 APC/Cy7 (clone 53-6.7) (1:400), CD3 FITC (clone 17A2) (1:300), CD45 BV510 (clone 30-F11) (1:300), CD44 BV421 (clone IM7) (1:400), PD-1 PE (clone 29F.1A12) (1:200), LAG3 PerCP-Cy5.5 (clone C9B7W) (1:100), and Tigit APC (clone1G9) (1:100). CD8 T cells were gated on live, CD3+/CD8+ double-positive cells. The cells were then further classified based on the expression of PD-1 and Lag-3. For the macrophage panel, cells were stained with CD45 BV510, F4/80 APC/Cy7 (clone BM8) (1:100), CD11b BV421 (clone M1/70) (1:600), CD64 PerCP-Cy5.5 (clone X54-5/7.1) (1:200), MERTK FITC (clone 2B10C42) (1:100), PD-L1 PE (clone 10F.9G2) (1:200), and Ly-6G APC/Cy7 (clone 1A8) (1:100). Live cells were gated for CD45+ cells. Neutrophils were gated by the expression of Ly-6Ghi/CD11b+, and not included in further analysis. Macrophages were gated as F4/80+/CD11bhi population and MERTKhi/CD64hi population. Monocytes were confirmed with two population gates as F4/80lo/CD11b+ and MERTKlo/CD64lo , as previously described79-82. For the tetramer panel, cells were stained with CD8 APC/Cy7, CD35 BV410, CD44 BV421, LAG3 PerCP Cy5.5, PD-1 APC (clone 29F.1A12) (1:100), CD4 FITC (clone GK1.5) (1:200), and MHC-I-SIINFEKL tetramer PE (NIH) (1:200). Live CD8+ cells were gated CD44hi/tetramer+. Cells were further analyzed for expression of PD-1 and Lag-3. IMs are identified based MERTKhi CD64hi CD11bhi, and subdivided into three pulmonary IM subtypes: IM1 (CD11clo CD206+ MHCIIlo), IM2 (CD11clo CD206+ MHCIIhi), and IM3 (CD11chi CD206lo MHCIIhi). All flow cytometry antibodies used were purchased from Biolegend unless otherwise indicated.

Validation

Pre-validated antibodies were purchased from well recognized vendors and reported by other researchers. We based specificity on their provided description and data sheets. Anti-PD-1 and isotype control were validated by the manufacturer, Bristol-Myers Squibb. Titrations were performed by lab personnel to determine optimal dilution concentrations.

Eukaryotic cell lines

Policy information about cell lines

Cell line source(s)

NA13 cell line was isolated and cultured from N-butyl-N-(4-hydroxybutyl) nitrosamine (BBN) carcinogen-induced bladder tumor of C57BL/6 female mice. E0771 was a gift from Dr. Traci Lyons (University of Colorado). B16F10 was obtained from the American Type Culture Collection (ATCC) through the University of Colorado Tissue Culture Core.

Authentication

Cell lines were authenticated by the University of Colorado Tissue Culture Core. Whole exome sequencing of NA13 was performed to confirm cell lineage and
characteristics, and is described in detail in Tu, et al., Science Advances 2019;5(2)

Mycoplasma contamination

All cell lines have been confirmed to be mycoplasma-free through STR DNA Profiling PowerPlex-16 HS Kit (Promega) by the University of Colorado Cancer Center Tissue Culture Shared Resource.

Commonly misidentified lines
(See ICLAC register)

N/A

Palaeontology and Archaeology

Specimen provenance

Provide provenance information for specimens and describe permits that were obtained for the work (including the name of the issuing authority, the date of issue, and any identifying information).

Specimen deposition

Indicate where the specimens have been deposited to permit free access by other researchers.

Dating methods

If new dates are provided, describe how they were obtained (e.g. collection, storage, sample pretreatment and measurement), where they were obtained (i.e. lab name), the calibration program and the protocol for quality assurance OR state that no new dates are provided.

☐ Tick this box to confirm that the raw and calibrated dates are available in the paper or in Supplementary Information.

Ethics oversight

Identify the organization(s) that approved or provided guidance on the study protocol, OR state that no ethical approval or guidance was required and explain why not.

Note that full information on the approval of the study protocol must also be provided in the manuscript.

Animals and other organisms

Policy information about studies involving animals; ARRIVE guidelines recommended for reporting animal research

Laboratory animals

Mus musculus, C67BL/6, female, ordered from Charles River. Mice were received at 6 weeks old and allowed to acclimate for at least one week in sterile micro isolator cages with constant temperature and humidity. Mice had free access to food and water.

Wild animals

Wild animals were not used in this study.

Field-collected samples

Field-collected samples were not used in this study.

Ethics oversight	Mice were housed in specific-pathogen-free conditions and cared for in accordance with US National Institutes of Health guidelines, and all procedures were approved by the University of Colorado Denver Animal Care and Use Committee and carried out according to approved protocols.

Note that full information on the approval of the study protocol must also be provided in the manuscript.

Human research participants

Policy information about studies involving human research participants

Population characteristics	*Describe the covariate-relevant population characteristics of the human research participants (e.g. age, gender, genotypic information, past and current diagnosis and treatment categories). If you filled out the behavioural & social sciences study design questions and have nothing to add here, write "See above."*
Recruitment	*Describe how participants were recruited. Outline any potential self-selection bias or other biases that may be present and how these are likely to impact results.*
Ethics oversight	*Identify the organization(s) that approved the study protocol.*

Note that full information on the approval of the study protocol must also be provided in the manuscript.

Clinical data

Policy information about clinical studies

All manuscripts should comply with the ICMJEguidelines for publication of clinical research and a completedCONSORT checklist must be included with all submissions.

Clinical trial registration	N/A
Study protocol	N/A
Data collection	N/A
Outcomes	N/A

Dual use research of concern

Policy information about dual use research of concern

Hazards

Could the accidental, deliberate or reckless misuse of agents or technologies generated in the work, or the application of information presented in the manuscript, pose a threat to:

No	Yes	
☒	☐	Public health
☒	☐	National security
☒	☐	Crops and/or livestock
☒	☐	Ecosystems
☒	☐	Any other significant area

Experiments of concern

Does the work involve any of these experiments of concern:

No	Yes	
☒	☐	Demonstrate how to render a vaccine ineffective
☒	☐	Confer resistance to therapeutically useful antibiotics or antiviral agents
☒	☐	Enhance the virulence of a pathogen or render a nonpathogen virulent
☒	☐	Increase transmissibility of a pathogen
☒	☐	Alter the host range of a pathogen
☒	☐	Enable evasion of diagnostic/detection modalities
☒	☐	Enable the weaponization of a biological agent or toxin
☒	☐	Any other potentially harmful combination of experiments and agents

ChIP-seq

Data deposition

☐ Confirm that both raw and final processed data have been deposited in a public database such as GEO.

☐ Confirm that you have deposited or provided access to graph files (e.g. BED files) for the called peaks.

Data access links<br>*May remain private before publication.*	*For "Initial submission" or "Revised version" documents, provide reviewer access links. For your "Final submission" document, provide a link to the deposited data.*
Files in database submission	*Provide a list of all files available in the database submission.*
Genome browser session<br>(e.g. UCSC)	*Provide a link to an anonymized genome browser session for "Initial submission" and "Revised version" documents only, to enable peer review. Write "no longer applicable" for "Final submission" documents.*

Methodology

Replicates	*Describe the experimental replicates, specifying number, type and replicate agreement.*
Sequencing depth	*Describe the sequencing depth for each experiment, providing the total number of reads, uniquely mapped reads, length of reads and whether they were paired- or single-end.*
Antibodies	*Describe the antibodies used for the ChIP-seq experiments; as applicable, provide supplier name, catalog number, clone name, and lot number.*
Peak calling parameters	*Specify the command line program and parameters used for read mapping and peak calling, including the ChIP, control and index files used.*
Data quality	*Describe the methods used to ensure data quality in full detail, including how many peaks are at FDR 5% and above 5-fold enrichment.*
Software	*Describe the software used to collect and analyze the ChIP-seq data. For custom code that has been deposited into a community repository, provide accession details.*

Flow Cytometry

Plots

Confirm that:

☒ The axis labels state the marker and fluorochrome used (e.g. CD4-FITC).

☒ The axis scales are clearly visible. Include numbers along axes only for bottom left plot of group (a 'group' is an analysis of identical markers).

☒ All plots are contour plots with outliers or pseudocolor plots.

☒ A numerical value for number of cells or percentage (with statistics) is provided.

Methodology

Sample preparation	Tumors were mechanically dissociated in Click's media in the absence of mercaptoethanol or L-glutamine (Irvine Scientific). Cells were digested for 1 hour at 37°C with 500 units/ml collagenase type II and IV and 20 µg/ml DNase (Worthington Biochemical). The digested tissue suspension was then filtered through a 100 µm strainer. Filtered cells were carefully layered into a centrifuge tube containing 5 ml Lympholyte-M (Cedarlane). The cells were centrifuged at 1500g for 20 min., then the interface lymphocyte layer was carefully removed. The cells were washed prior to staining.
Instrument	All samples were run on the CyAn ADP flow cytometer, acquired using Summit software.
Software	Analysis was performed using FlowJo software.
Cell population abundance	Cell samples were not subjected to cell sorting. Immune populations were determined through singlet, live-dead, and CD45+ cell gating.
Gating strategy	Singlet, live cells were gated for CD45+ cells. CD8 T cells were gated on live, CD3+/CD8+ double-positive cells. The cells were then further classified based on the expression of PD-1 and Lag-3. Neutrophils were gated by the expression of Ly-6Ghi/CD11b+. Macrophages were gated as F4/80+/CD11bhi population and MERTKhi/CD64hi population. Monocytes were confirmed with two population gates as F4/80lo/CD11b+ and MERTKlo/CD64+. Cells were further analyzed for expression of PD-1 and Lag-3. For CD4 T cell analysis, singlet, live, CD45+ were gated for the CD4+/CD8- population. Further classification of activated CD4 T cells as CD25+/FoxP3- and regulatory CD4 T cells as FoxP3+ was performed. Samples of the flow gating strategy are provided in the Supplementary.

☒ Tick this box to confirm that a figure exemplifying the gating strategy is provided in the Supplementary Information.

Magnetic resonance imaging

Experimental design

Design type — *Indicate task or resting state; event-related or block design.*

Design specifications — *Specify the number of blocks, trials or experimental units per session and/or subject, and specify the length of each trial or block (if trials are blocked) and interval between trials.*

Behavioral performance measures — *State number and/or type of variables recorded (e.g. correct button press, response time) and what statistics were used to establish that the subjects were performing the task as expected (e.g. mean, range, and/or standard deviation across subjects).*

Acquisition

Imaging type(s) — *Specify: functional, structural, diffusion, perfusion.*

Field strength — *Specify in Tesla*

Sequence & imaging parameters — *Specify the pulse sequence type (gradient echo, spin echo, etc.), imaging type (EPI, spiral, etc.), field of view, matrix size, slice thickness, orientation and TE/TR/flip angle.*

Area of acquisition — *State whether a whole brain scan was used OR define the area of acquisition, describing how the region was determined.*

Diffusion MRI ☐ Used ☐ Not used

Preprocessing

Preprocessing software — *Provide detail on software version and revision number and on specific parameters (model/functions, brain extraction, segmentation, smoothing kernel size, etc.).*

Normalization — *If data were normalized/standardized, describe the approach(es): specify linear or non-linear and define image types used for transformation OR indicate that data were not normalized and explain rationale for lack of normalization.*

Normalization template — *Describe the template used for normalization/transformation, specifying subject space or group standardized space (e.g. original Talairach, MNI305, ICBM152) OR indicate that the data were not normalized.*

Noise and artifact removal — *Describe your procedure(s) for artifact and structured noise removal, specifying motion parameters, tissue signals and physiological signals (heart rate, respiration).*

Volume censoring — *Define your software and/or method and criteria for volume censoring, and state the extent of such censoring.*

Statistical modeling & inference

Model type and settings — *Specify type (mass univariate, multivariate, RSA, predictive, etc.) and describe essential details of the model at the first and second levels (e.g. fixed, random or mixed effects; drift or auto-correlation).*

Effect(s) tested — *Define precise effect in terms of the task or stimulus conditions instead of psychological concepts and indicate whether ANOVA or factorial designs were used.*

Specify type of analysis: ☐ Whole brain ☐ ROI-based ☐ Both

Statistic type for inference (See Eklund et al. 2016) — *Specify voxel-wise or cluster-wise and report all relevant parameters for cluster-wise methods.*

Correction — *Describe the type of correction and how it is obtained for multiple comparisons (e.g. FWE, FDR, permutation or Monte Carlo).*

Models & analysis

n/a	Involved in the study
☐	☐ Functional and/or effective connectivity
☐	☐ Graph analysis
☐	☐ Multivariate modeling or predictive analysis

Functional and/or effective connectivity — *Report the measures of dependence used and the model details (e.g. Pearson correlation, partial correlation, mutual information).*

Graph analysis — *Report the dependent variable and connectivity measure, specifying weighted graph or binarized graph, subject- or group-level, and the global and/or node summaries used (e.g. clustering coefficient, efficiency, etc.).*

Multivariate modeling and predictive analysis

Specify independent variables, features extraction and dimension reduction, model, training and evaluation metrics.
